# Sharp Bounds and Normalization of Wiener-Type Indices

**DOI:** 10.1371/journal.pone.0078448

**Published:** 2013-11-08

**Authors:** Dechao Tian, Kwok Pui Choi

**Affiliations:** 1 Department of Statistics and Applied Probability, National University of Singapore, Singapore, Singapore; 2 Department of Mathematics, National University of Singapore, Singapore, Singapore; University of East Piedmont, Italy

## Abstract

Complex networks abound in physical, biological and social sciences. Quantifying a network’s topological structure facilitates network exploration and analysis, and network comparison, clustering and classification. A number of Wiener type indices have recently been incorporated as distance-based descriptors of complex networks, such as the R package QuACN. Wiener type indices are known to depend both on the network’s number of nodes and topology. To apply these indices to measure similarity of networks of different numbers of nodes, normalization of these indices is needed to correct the effect of the number of nodes in a network. This paper aims to fill this gap. Moreover, we introduce an 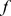-Wiener index of network 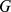, denoted by 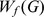. This notion generalizes the Wiener index to a very wide class of Wiener type indices including all known Wiener type indices. We identify the maximum and minimum of 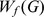 over a set of networks with 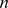 nodes. We then introduce our normalized-version of 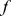-Wiener index. The normalized 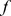-Wiener indices were demonstrated, in a number of experiments, to improve significantly the hierarchical clustering over the non-normalized counterparts.

## Introduction

Recent years witness exponential growth of available biological network data. Thanks to past decades’ breakthrough in biotechnology, researchers now are able to interrogate molecular interactions at systems level. It has since been observed that topological properties of these networks provide important insight into the functions of proteins, and their relationship with one another [1]–[8]. For examples, degree distribution, average clustering coefficient, diameter, centrality, lethality and graphlet distribution have been extensively studied. Hopefully, based on a carefully chosen list of network topological properties and methods in quantifying them, a complex network is adequately summarized in the form of a numerical 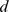-dimensional vector where 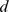 is the number of topological properties in consideration. This representation enables us to take full advantage of a host of classification and clustering techniques to compare complex networks.

A significant step towards this direction is facilitated by the introduction of the R package QuACN by Mueller et al. [9]. QuACN computes the values of different categories of descriptors in a network. One such category is the distance-based descriptors which include Wiener index, Harary index, etc. The use of Wiener index and related type of indices dates back to the seminal work of Wiener in 1947 [10], [11]. Wiener introduced his celebrated index to predict the physical properties, such as boiling point, heats of isomerization and differences in heats of vaporization, of isomers of paraffin by their chemical structures. Viewing the chemical structure of an isomer as a connected graph, the Wiener index is defined as 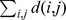 where 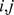 represent nodes in the graph, 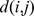 the distance between nodes 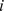 and 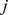 which is defined as the length of a shortest path between them, and the sum is over all pairs of nodes in the graph. Wiener index has since inspired many distance-based descriptors in Chemometrics. These include Harary index [12], hyper Wiener index [13], q-analog of Wiener index [14], Wiener polynomial [15], Q-index [16], Balaban J index [17], and information indices [18]–[20]. These indices, or commonly called descriptors, play significant roles in quantitative structure-activity relationship/quantitative structure-property relationship (QSAR/QSPR) models [21].

It is known that the Wiener type indices depend both on a network’s number of nodes and its topology. When the numbers of nodes in the networks are equal, as in the applications to isomers, these indices provide informative measures of the branching property of the networks and hence a fair comparison among them. However, when they are used to measure similarities of networks with different numbers of nodes, the intended measure of topological structures will be masked by the sizes of the networks. Normalization of a Wiener type index expectedly minimizes the effect of the network’s number of nodes and hence brings forth its topological structure better. Furthermore, it is also desirable for the normalized index to take value in an absolute scale for better understanding and interpretation. This paper seeks to fill this gap. The normalization introduced in definition 2 below fulfils this purpose. This definition will be of limited practical value if the sharp upper and lower bounds of the index on a graph cannot be found explicitly. The objective of this article is three-fold. First, introduce a very general Wiener type index. We call it 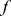-Wiener index, and denote it by 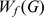 for a graph 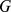. This definition includes all known Wiener type indices as special cases. Second, identify the maximum and minimum values of 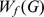 over a class of connected networks 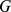 or a class of connected trees 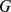. We are able to derive explicit formulas for these optimal values. Third, propose a normalized version, 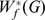 which takes value in 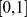 for better interpretation and network comparison.

This paper is organized as follows. We first introduce some standard graph-theoretic notations and recall some special graphs. We then introduce the functional analog of Wiener index, 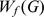, and our proposed normalized versions of this functional Wiener index in the method section. In the result section, we provide our main results Theorems 1 to 4. Theorem 1 gives the maximum and the minimum of 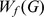 over the set of connected graphs of 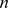 nodes, and characterization of graphs achieving the maximum or the minimum. Theorem 2 gives a parallel result when the maximum and minimum are taken over the set of connected trees of 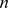 nodes. Theorem 3, (respectively Theorem 4) identifies the maximum of 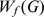 over the set of connected graphs (respectively connected trees) of 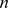 nodes with specified maximum degree. We also give a brief description of related works in next section. Then, we consider special cases of 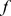 in 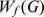 to provide explicit expressions of the maximum and the minimum of Wiener, Harary, hyper Wiener, generalized Wiener indices. In the experiment section, we report the performance of hierarchical clustering based on the usual Wiener type indices and the normalized version of these in our experiments. We end with conclusions section of this paper.

## Methods

### Definitions and Terminologies

Let 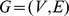 be a simple (that is, no self-loops nor multiple edges) connected graph on 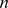 nodes where 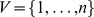 and 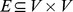. Denote by 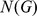 as the number of nodes in 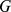. Let 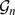 denote the set of all simple, connected graphs with 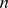 nodes. A graph having no cycles is called a tree, and we let 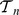 denote the set of all connected trees with 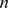 nodes. The distance 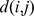 between any pair of nodes, 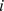 and 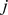, in 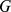 is the number of edges in a shortest path from 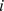 to 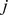. Let 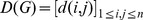 be the distance matrix. We denote the maximum degree of 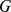 by 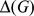.

**Figure 1:**
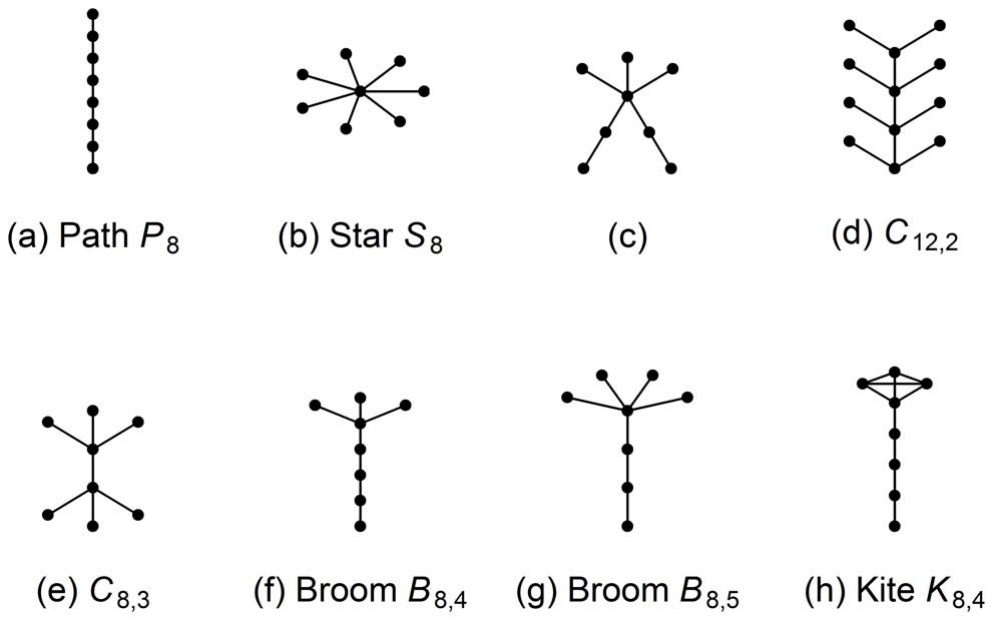
Some special graphs. **Figure 1** (a) to (g) are trees.

Figure 1 shows some special graphs we frequently refer to in this paper. A path graph, 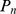, is a graph that can be drawn so that all of its vertices and edges lie on a straight line. Figure 1(a) shows 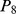. A star, 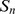, is a tree with one internal node and 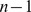 leaves. 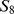 is shown in Figure 1(b). A complete graph, 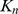, is a graph with 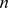 nodes in which every pair of distinct nodes is connected by an edge. A caterpillar, 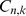, is a tree with a central path with number of nodes 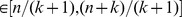 where at most one end node of the central path has less than 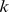 leaves, each of the other nodes in the central path has 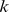 leaves. Figures 1(d) and 1(e) show caterpillars 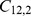 and 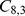 respectively. A broom 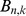 is a tree joining a star 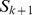 and a path 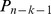 by attaching a pendant node (or leaf) in 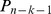 to a pendant node of 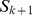. For examples, brooms 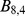 and 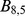 are shown in Figures 1(f) and 1(g) respectively. A kite 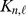 is a graph obtained from connecting two end nodes one from a complete graph 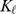 and one from a path 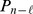. Figure 1(h) shows a kite 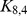.

Throughout this paper, 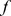 denotes a monotone function defined on nonnegative integers. We define a functional-analog Wiener index below. Our definition contains the Wiener index, Harary index, hyper Wiener index, compactness, average efficiency, generalized Wiener index, Wiener polynomial, 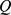-index, 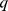-analogy of Wiener index as special cases. For detail, see subsection Important special cases. We abbreviate it as 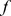-Wiener index. Thanks to an anonymous reviewer of this article, this definition has also been independently introduced by Schmuck et al. [22].

**Definition 1.** *The* 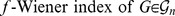-Wiener index of 
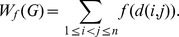
 *is defined by*

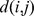



*Here* 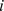 *denotes the shortest distance between nodes* 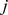 *and* 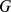.

The number of nodes of 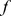 has a very strong effect on Wiener type indices (see Results section). In order to apply 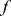-Wiener index for comparing networks, which often differ in the numbers of nodes, we are led to propose a normalized version for graphs and a normalized version for trees for better interpretation of the index.

**Definition 2.** *(a) The normalized* 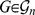 *-Wiener index for a graph* 
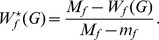
 *is defined as*

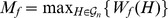



*Here* 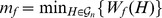 *and* 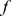.

*(b) The normalized* 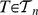 *-Wiener index for a tree* 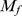 *is similarly defined where the maximum* 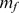 *and the minimum* 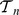 *are taken over* 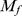 *instead.*

These normalized versions will be of limited practical value if one cannot compute 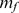 nor 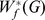. Our main results, stated in Theorems 1 and 2, show that these optimal upper and lower bounds can be easily computed. Moreover, they characterize those graphs which attain the maximum or the minimum.

By definition, 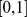 takes values in 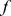. When 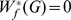 is a non-decreasing function, Theorem 1 below shows that 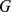 if and only if 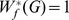 is a path graph, and 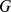 if and only if 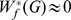 is a complete graph. So 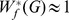 (respectively, 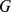) suggests 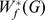 looks like a path graph (respectively, a complete graph). And hence the numerical value of 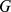 provides an indication how 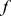 is like.

### Effect of Number of Nodes on Wiener Type Indices

It is known that the Wiener index for a connected graph with 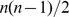 nodes ranges from 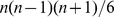 to 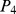 (see Corollary 5 below or [23]–[25] ). This wide range can be undesirable if it is used for comparing similarity of graphs with different number of nodes. For example, consider two path graphs, 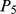 and 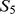, with 4 nodes and 5 nodes respectively, and a star graph with 5 nodes, 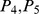. Values of the Wiener index for 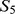 and 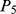 are respectively 10, 20 and 16, giving the false impression that 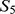 and 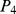 are more similar than that of 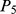 and 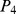. However, values of the normalized Wiener index are 0 for 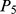 and 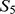, and 1 for 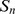. This example is far from being an isolated case, it can be shown that if the number of nodes of a path graph is at least 26% more than the number of nodes in another path graph, there exists a star graph whose Wiener index is closer to that of the path graph with smaller number of nodes.

The normalized Wiener index of 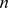, star with 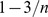 nodes, is 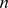, suggesting stars of sufficiently large 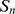, based on the normalized Wiener index, 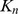 is very similar to a complete graph. This is concordant with the fact that a 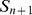 is the line graph of 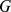 [26].

### Main Idea

A key ingredient in our proofs is a matrix majorization (see Supporting information file Text S1 for definition) argument. Given a connected graph 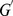, we can transform it to another graph 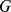 such that the distance matrix of 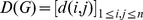, 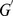 majorizes the corresponding distance matrix of 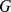. Since Wiener index of 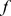, or its generalization 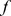-Wiener index for increasing function 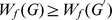, is the sum of the upper diagonal entries in the distance matrix, it follows that 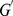. The construction of 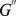 is fairly straightforward as can be seen in the proofs. The construction of 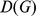 such that 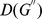 is majorized by 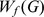 requires delicate and judicious pruning and regrafting. However, the essential idea remains the same. Technical details of proofs are given in supporting information file Text S1.

## Results

We provide explicit expressions for the maximum and minimum of 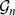 over 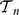, and over 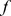 in Theorems 1 and 2 below. We also characterize those graphs or trees attaining the extremum. Theorems 3 and 4 concern trees or graphs with a specified maximum degree. For simplicity of presentations, we shall only state our results for non-decreasing function 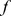. Analogous results for non-increasing 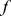 can be deduced easily by replacing 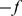 by 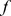.

**Theorem 1** *Let* 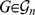 *be a non-decreasing function on nonnegative integers, and* 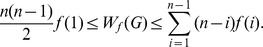 *, then*

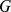



*The lower bound is attained if and only if* 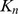 *is* 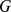 *. The upper bound is attained if and only if* 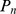 *is* 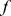.

**Theorem 2** *Let* 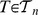 *be a non-decreasing function on nonnegative integers, and* 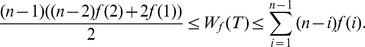 *, then*

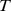



*The lower bound is attained if and only if* 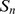 *is* 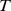 *. The upper bound is attained if and only if* 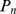 *is* 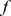.

**Theorem 3** *Let* 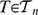 *be a non-decreasing function on nonnegative integers. Then, for any* 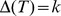 *with* 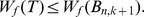 *, we have*

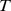



*The upper bound is attained if and only if* 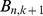 *is a broom* 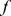.

**Theorem 4** *Let* 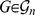 *be a non-decreasing function on nonnegative integers. Then, for any* 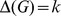 *with* 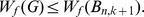 *, we have*

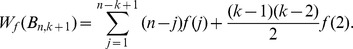



*Moreover,*

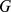



*Equality holds if and only if* 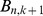 *is* 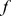.

## Related Work

The proofs of Theorems 1 to 4 will be given in supporting information file Text S1. Theorem 2 has also been independently obtained by Wagner et al. (see Theorem 2.7 and Corollary 4.1 in [27]). Special cases of Theorems 1 to 4 for particular Wiener type index are known in the literature. For examples, the complete graph (respectively, the path graph) is shown to be the minimizer (respectively, maximizer) of the Wiener index among simple connected graphs with the same number of nodes in [23]–[25]. Similar conclusions are proved to hold for the hyper Wiener index in [25], and the Harary index in [28]. The results in Theorems 1 to 4 in its full generality as 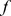-Wiener index are novel to the best knowledge of the authors. Moreover, we have provided a unifying methodology for the proofs.

### Important Special Cases

Since its introduction, Wiener index has inspired many variants and thoroughly studied in a sizeable literature [29]. By choosing appropriate functions 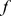, the 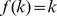-Wiener index can be reduced to a number of commonly used descriptors as follows.

If we take 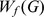, 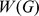 written as 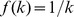 is the well-studied descriptor introduced by Wiener in 1947 [10], [11].

Taking 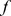, the 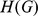-Wiener index is the Harary index [12], denoted by 
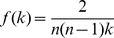
 which is shown to be more discriminating than the Wiener index [12]. Latora and Marchiori in 2001 [30], used a scaled version of the Harary index (more precisely, 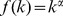) to measure a network’s efficiency in information exchange.

Taking 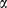, where 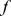 can be positive or negative, the 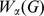-Wiener index is called generalized Wiener index, denoted by 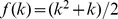 [31].

If 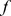, the 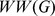-Wiener index is known as the hyper Wiener index [13], denoted by 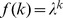.

Taking 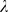, where 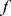 is regarded as a parameter, the 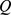-Wiener index is called the Hosoya polynomial or Wiener polynomial [15]. With an additional factor 2, the Hosoya polynomial is called 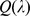-index and denoted by 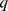 in [16].

The 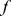-analog of the Wiener index, introduced by Zhang et al. (2012) in [14] is simply the 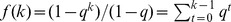-Wiener index by choosing 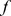.

## Applications

By specializing 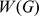 to various forms in Theorems 1 and 2, we provide below explicit sharp upper bounds and sharp lower bounds for the Wiener index 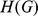, the Harary index 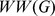, the hyper Wiener index 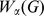, and the generalized Wiener index 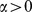 for 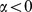 and 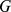.

**Corollary 5** *Let* 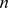 *be a simple, connected graph with* 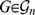 *nodes (that is,* 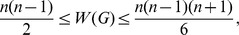 *), we have*

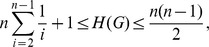


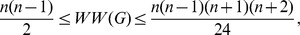


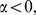
when 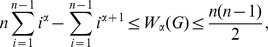


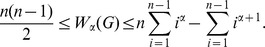
when *α*>0,
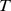



**Corollary 6** *Let* 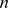 *be a tree with* 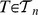 *nodes (that is,* 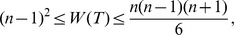 *), we have*

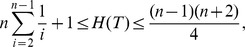


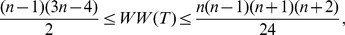


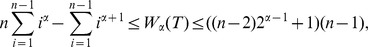
when *α*<0,
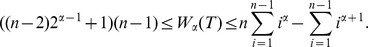
when *α*>0,
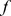



## Experiments

We describe below three experiments to compare the hierarchical clustering using normalized 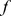-Wiener indices with the hierarchical clustering using non-normalized 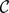-Wiener indices. Each experiments consists of 3 main steps.

Step 1: A collection of networks (or graphs) or trees, 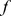, are chosen to be clustered. The collection is detailed in each experiment below.

Step 2: Seven functions are chosen to form the 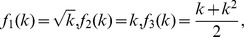-Wiener indices. In all our experiments, we choose



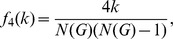
and



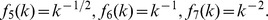


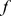



The first four functions chosen are increasing and the 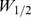-Wiener indices correspond to the usual 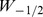 index, Wiener index, the hyper Wiener index and the compactness index. The remaining 3 functions chosen are decreasing and correspond to the 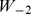 index, the Harary index and the 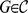 index. Hopefully these indices collectively capture some essential characters of networks and useful for clustering. For 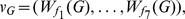, we construct two characteristic vectors,
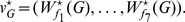


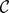



Step 3: We adopt a clustering algorithm to cluster 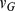 using 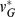 and then produce a dendrogram. We do the same using 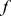. Minimum variance method algorithm due to Ward [32] which is made available in R base package [33], was used in all the experiments. The computed the Adjusted Rand Index (ARI) in all the experiments are summarized in Table 1 below.

**Table 1:** Adjusted Rand Index (ARI) for clustering (or classification) of networks in our three experiments.

	Non-normalized	Normalized
Experiment 1.1	0.44 (0.02)	0.88 (0.07)
Experiment 1.2	0.41 (0.06)	1.00 (0.01)
Experiment 1.3	0.38 (0.10)	1.00 (0.00)
Experiment 1.4	0.36 (0.11)	0.97 (0.10)
Experiment 1.5	0.30 (0.12)	0.62 (0.07)
Experiment 2	0.10	1.00
Experiment 3	0.04	0.86

For experiments 1.1 to 1.5, we report the mean and the standard deviation (number in parenthesis) of ARI. Mean and standard deviation of ARI for experiments 1.1 to 1.5 under random clustering are 0 and 0.05 respectively.

### Experiment 1: Hierarchical Clustering of Random Networks

**Figure 2:**
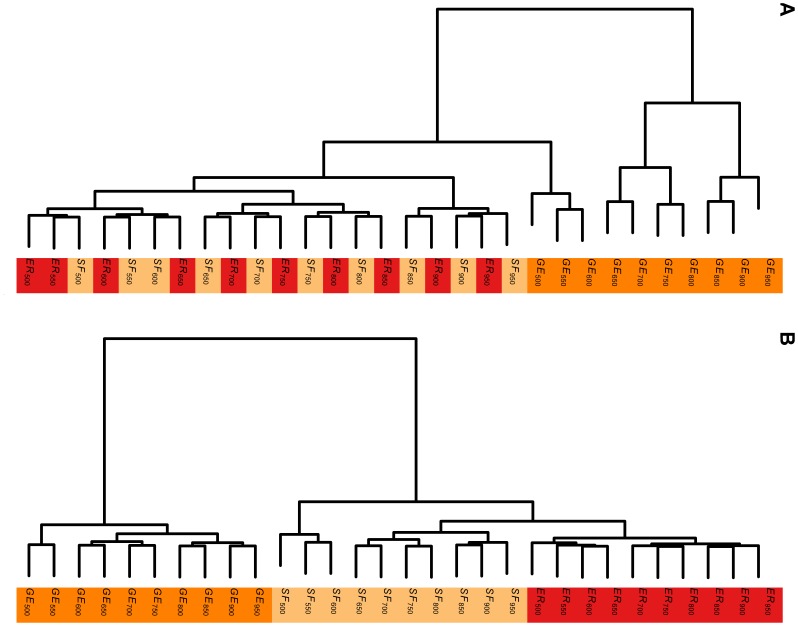
Hierarchical clustering of random networks. 30 networks with 10 each generated by the Erdos-Renyi (ER), scale-free (SF) and geometric (GE) random network models. Panel (A) shows the hierarchical clustering based on the 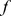-Wiener indices (see Step 1 on page 8 for functions used). The adjusted rand index (ARI) for this clustering is 0.24. Panel (B) is the hierarchical clustering based on the normalized versions of the same 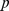-Wiener indices. The ARI of this clustering is 0.67. Number of nodes chosen are 500, 550, …, 950, and 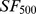 is 0.05 in the Erdos-Renyi model. A scale-free network with 500 nodes is denoted by 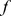. The others are denoted in a similar way.

**Figure 3:**
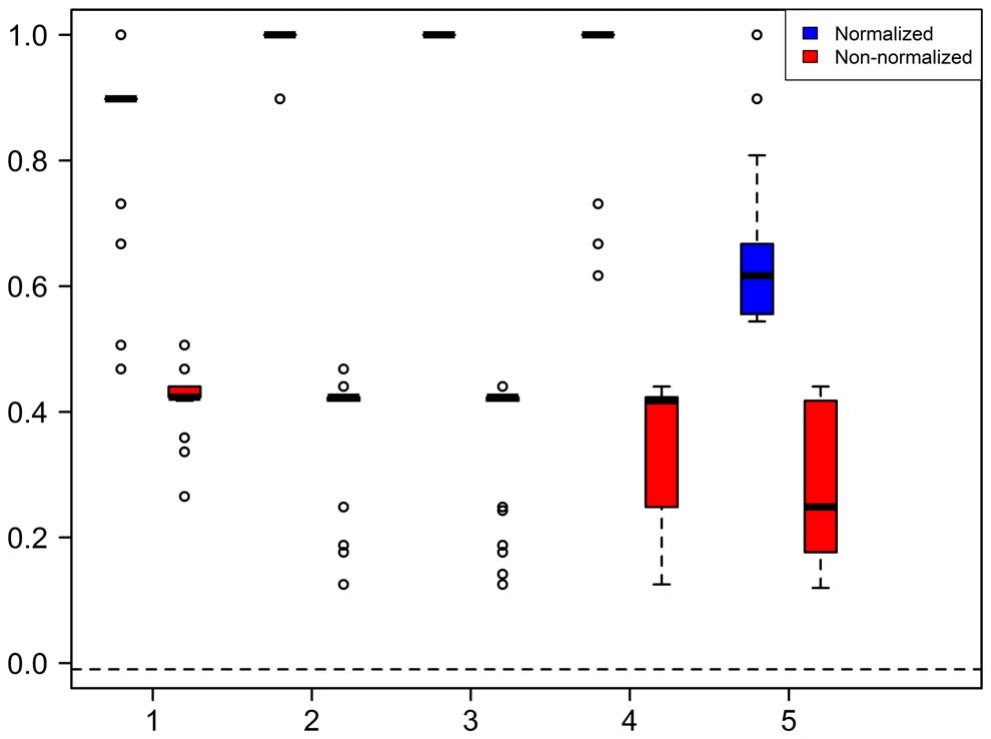
Boxplots of adjusted rand index for measuring the extent of agreement of clustering of the random networks using non-normalized 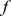
 **-Wiener indices versus normalized** 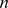 **-Wiener indices.**

The collection of networks chosen for this experiment is the networks generated by some commonly used random network models, namely, Erdos-Renyi (ER) model [34], [35], scale-free (SF) network model [36] and 3-D geometric model (GE) [37]. Each of these random network models is applied to generate 10 random networks with the number of nodes ranging from 500 to 950 with step of increment 50. Experiment 1 consists of 5 small, but similar, experiments. We enumerate these 5 small experiments as 1.1,…, 1.5. Subsection after experiments provides more details on how to generate these random networks. We then apply Steps 2 and 3 above to form two dendrograms: one using 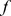-Wiener indices without normalization (Figure 2A) and the other dendrogram using normalized 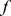-Wiener indices (Figure 2B). To quantify the classification of the two methods: with and without normalization, we adopt the commonly used Adjusted Rand Index (ARI) [38] for classification validation. ARI measures the accuracy of classification, and takes values between −1 and 1. The larger the ARI is, the better is the classification. The ARI for Figures 2A and 2B are respectively 0.18 and 0.56 for Experiment 1.5. Using normalized 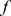-Wiener indices lead to a substantial improvement in the classification. We repeat Experiments 1.1 to 1.5 1000 times each. The boxplots of the ARI are shown in Figure 3. The means and standard deviations for these experiments are given in Table 1. They clearly demonstrate the superiority of classification using normalized 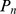-Wiener indices.

### Experiment 2: Hierarchical Clustering of Trees

The collection of trees to be classified consists of 10 paths (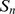), 10 stars (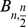), 10 brooms (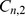), 20 caterpillars (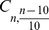 which is like a path, and 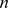 which is like a star), and for 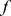 ranging from 500 to 950 with step of increment 50.

**Figure 4:**
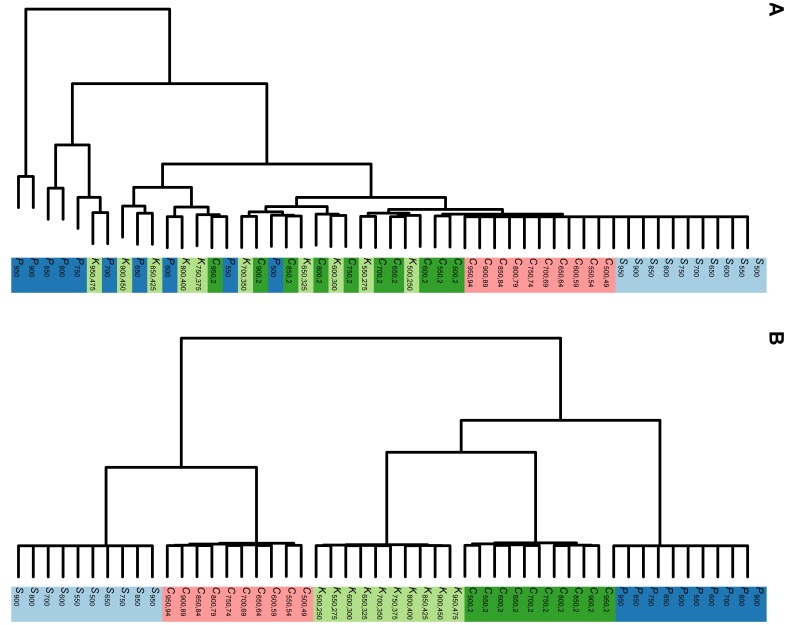
Hierarchical clustering of trees. Panel (A) shows the hierarchical clustering based on the 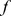-Wiener indices (see Step 1 on page 6 for functions used). The adjusted rand index (ARI) is 0.1. Panel (B) shows the hierarchical clustering based on normalized 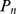-Wiener indices. The ARI is 1. Trees used in the clustering consist of paths (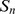), stars (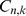), caterpillar-like trees (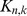), kites (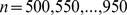). Number of nodes 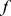.

Figure 4 shows the two dendrograms. The ARI for Figures 4A and 4B are respectively 0.10 and 1.00. This demonstrates that using normalized 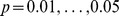-Wiener indices provides much better accuracy for classification purposes. The result in this experiment is consistent with that of experiment 1.

### Experiment 3: Hierarchical Clustering of Random Networks and Trees

**Figure 5:**
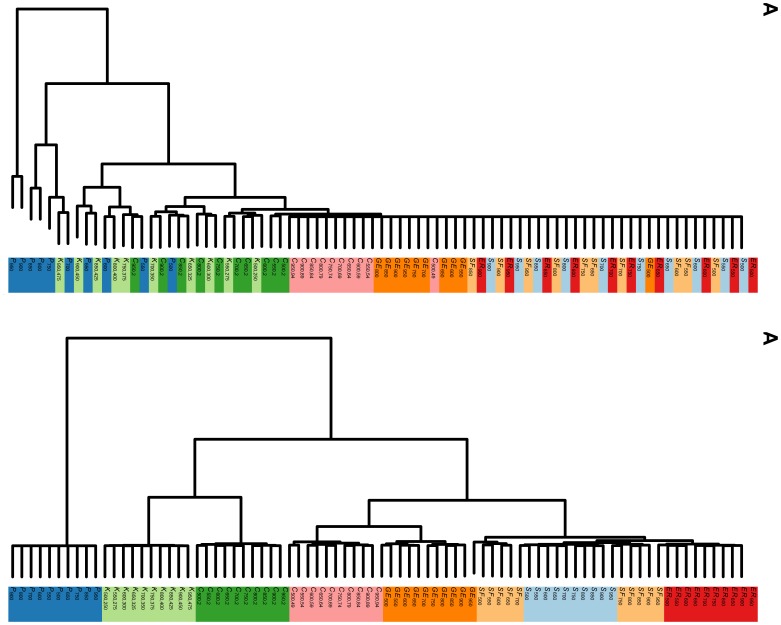
Hierarchical clusters of trees and graphs. Panel (A) shows the hierarchical clustering based on the 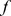-Wiener indices (see Step 1 on page 6 for functions used). The adjusted rand index (ARI) is 0.04. Panel (B) shows the hierarchical clustering based on normalized 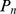-Wiener indices, and ARI = 0.86. Trees used are paths (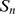), stars (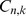), caterpillar-like trees (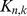), kites (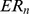). Graphs are generated by Erdos-Renyi (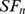), scale-free (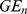) and geometric (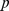) random network models. The parameter, 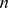, in the Erods-Renyi random graph equals to 0.05, number of nodes 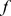 = 500,550,…,950.

The collection of networks consists of (i) networks generated by three random network models, namely, ER model, SF Model and 3-D geometric model; (ii) some trees such as paths, brooms, caterpillars, stars. Figure 5 shows the two dendrograms formed. And the ARI for Figures 5A and 5B are respectively 0.04 and 0.86.

### Details on Generating Random Networks

We describe here in details on how to choose the networks generated by the three random network models in experiments 1 and 3.

Experiment 1 consists of 5 small, but similar, experiments which we label as Experiment 1.1, …, Experiment 1.5 which correspond to 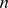 respectively. Now we describe Experiment 1.5 in details.

### ER Model

There are two parameters in the ER model, namely, 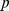, the number of nodes, and 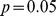, the probability that an edge is formed between a pair of nodes. All edges are formed independently of each other. In Experiment 1.5, where 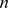, we choose 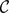 ranging from 500 to 950 with step of increment 50. We generate an ER network using ‘erdos.renyi.game’ function available in the R package igraph [39]. If the network is connected, we keep it in 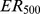 and denote it as 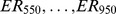. If not, then we repeat the function ‘erdos.renyi.game’ until a connected network is obtained. Similarly, 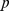 are generated.

### SF Model

We also construct ten SF networks by the function ‘barabasi.game’ available in the R igraph package. We shall describe how to grow a SF network with 500 nodes for a given 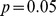, say 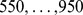. The other 9 SF networks with 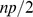 nodes are constructed in a similar manner. In ‘barabasi.game’ function, we set number of vertices 500, number of edges to be added in each time step 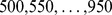 rounded to the nearest integer, and the option to create a directed graph false.

### Geometric Model

We generate ten 3-D geometric networks with 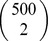 nodes. We shall describe how to construct one with 500 nodes as follows. The rest are constructed similarly. We first place 500 nodes in a unit cube uniformly and independently, then we compute all the 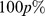 pairwise distances and rank these distances in ascending order. We choose the top 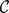 of these pairwise distances and connect their corresponding nodes. If this network is connected, then we keep it in 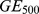 and denote it by 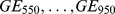. Otherwise, we discard it, and repeat the above procedure until we get a connected network. The other networks 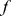 are constructed similarly.

## Conclusions

Wiener index and other Wiener type indices have been commonly applied in Chemometrics to associate structures and physicochemical properties of molecules. Recently, these indices are incorporated in quantifying complex networks as in QuACN [9] and NetCAD [40]. In this article, we first generalize Wiener index to a general functional form, called 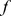-Wiener index. This 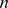-Wiener index contains all well-known Wiener type indices as special cases such as Wiener index, Harary index, hyper Wiener index, compactness, and average efficiency. We provide a unifying method to identify the maximum and minimum over the set of simple connected graphs with 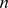 nodes, or the set of simple connected trees with 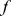 nodes (Theorems 1 and 2). Explicit sharp upper and lower bounds for Wiener index, Harary index, hyper Wiener index and the generalized index are deduced over networks (Corollary 5) and over trees (Corollary 6). Moreover, the maximizer and minimizer are characterized in Theorems 1 and 2. We believe these results are general and of independent interests.

Armed with these maximum and minimum values, we propose a normalized version of 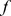-Wiener index over networks, and a similar version over trees. These normalized versions provide better interpretation of indices over networks of varying number of nodes than the non-normalized one. We conduct a number of experiments to compare the clustering performance using normalized 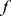-Wiener indices with that of the non-normalized 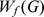-Wiener indices. The results of these experiments consistently demonstrate that using normalized versions improved clustering substantially. The normalized versions capture similar topological structures among networks with different number of nodes better. Our method of optimizing 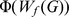 can be easily extended to index of the form 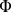 where 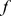 and 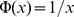 are monotone functions. For example, taking 
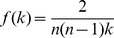
 and 
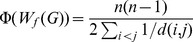
 leads to 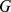 which measures small-world behvaior of network 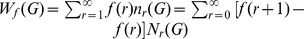 [8]. For other descriptors, it is of interest to study whether normalization is needed; if so, how best to normalize them; and to what extent normalization improve network comparison.

Observe that 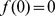 where we assume 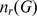, 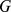 denotes the number of pairs of nodes in 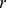 with distance equals 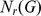, and 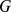 the number of pairs of nodes in 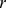 with distance greater than 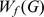. Since in most biological networks the number of nodes is large, one may normalize a scaled-version of 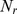 in terms of the asymptotic distribution of the 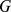
’s under the assumption that the observed network 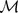 is generated by a given random network model 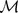. This will enable us to determine the likelihood that the observed network is generated by 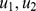. Currently a fair amount of information about shortest paths in some network models is available in [41], [42]. How to make use of these results seems like a worthwhile future project.

## Supporting Information

Figure S1Illustrating the choices of 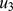 and 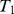 in Lemma 2. Here 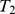 has 5 nodes, 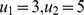 3 nodes. We choose 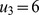 and 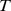. Tree 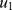 is constructed by joining 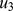 and 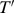 while 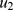 by joining 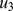 and 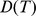. 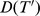 and 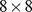 are 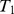 matrices where the first 5 columns correspondent to the 5 nodes in 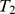, and the last 3 rows correspondent to the 3 nodes in 
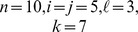
.(TIF)

Figure S2Illustration of Lemma 3. Here 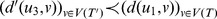. From the counts of the distances above, it is clear that 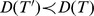 and 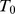.(TIF)

Figure S3Illustration of the subtree pruning and regrafting algorithm. Here 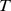 is obtained from 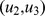 first by deleting the edge 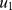 and then connecting 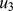 and 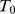. 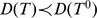 is proved to satisfy these properties: (i) 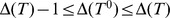; (ii) 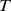; and (iii) number of pendant nodes is one less than that of 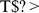.(TIF)

Text S1 Detailed proof for Theorems 1–4.
(PDF)
