# Correction: Sharp Bounds and Normalization of Wiener-Type Indices

**DOI:** 10.1371/journal.pone.0095652

**Published:** 2014-04-15

**Authors:** The PLOS ONE Staff

## Notice of Republication

This article was republished on March 18, 2014, to include the “Text_S1” file in the supporting information. Please download this article again to view the correct version. The originally published, uncorrected article and the republished, corrected article are provided here for reference.

## Supporting Information

File S1 Originally published, uncorrected article.

File S2 Republished corrected article.
