# Supplementary figures and images for: Sharp Bounds and Normalization of Wiener-Type Indices
Source: PLoS One. 2013 Nov 8;8(11):e78448. doi: 10.1371/journal.pone.0078448 (PMC3832646; doi:10.1371/journal.pone.0078448)

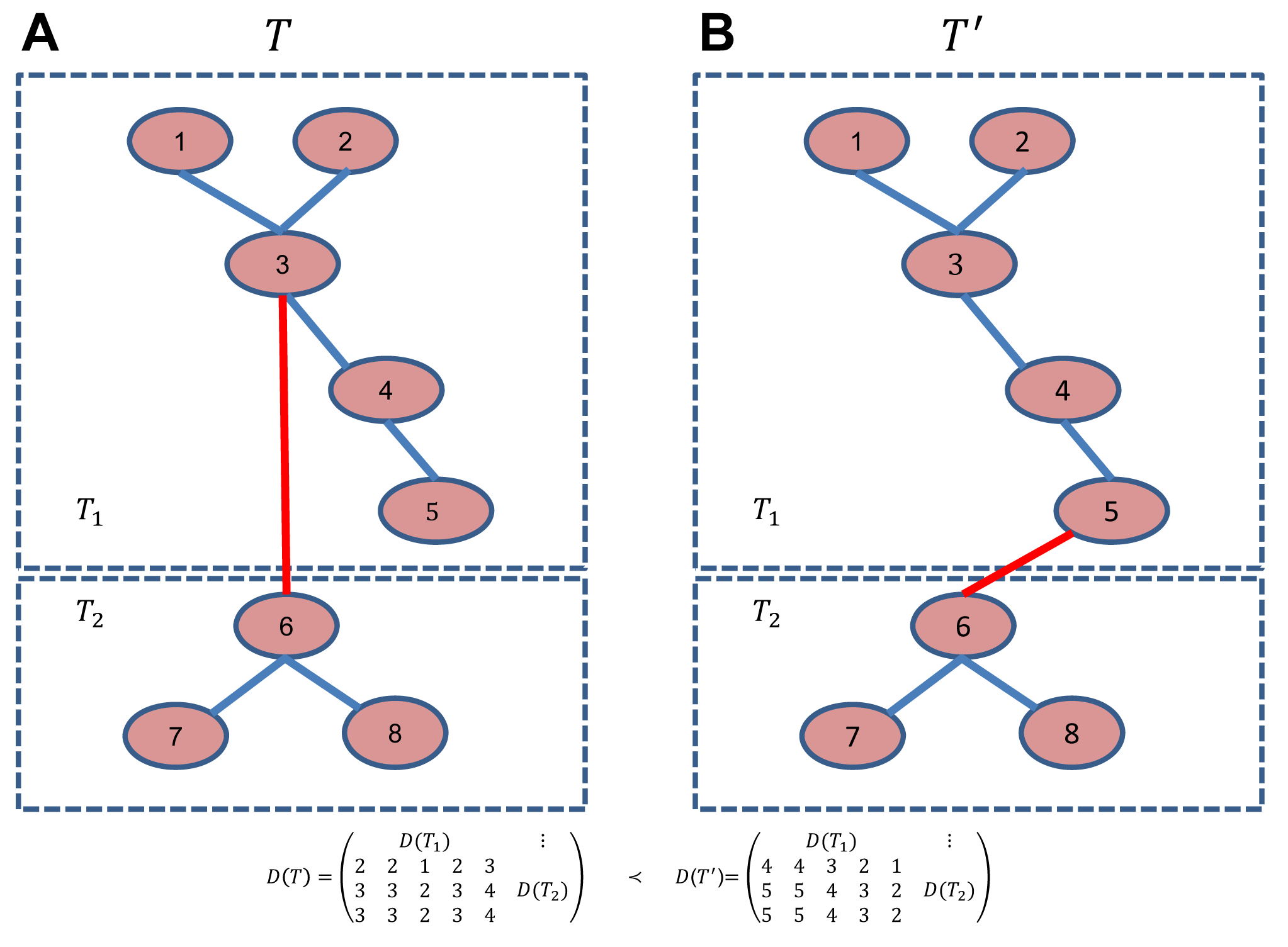

Supplement: Figure S1 — Illustrating the choices of and in Lemma 2. Here has 5 nodes, 3 nodes. We choose and . Tree is constructed by joining and while by joining and . and are matrices where the first 5 columns correspondent to the 5 nodes in , and the last 3 rows correspondent to the 3 nodes in . (TIF) [file pone.0078448.s001.tif]

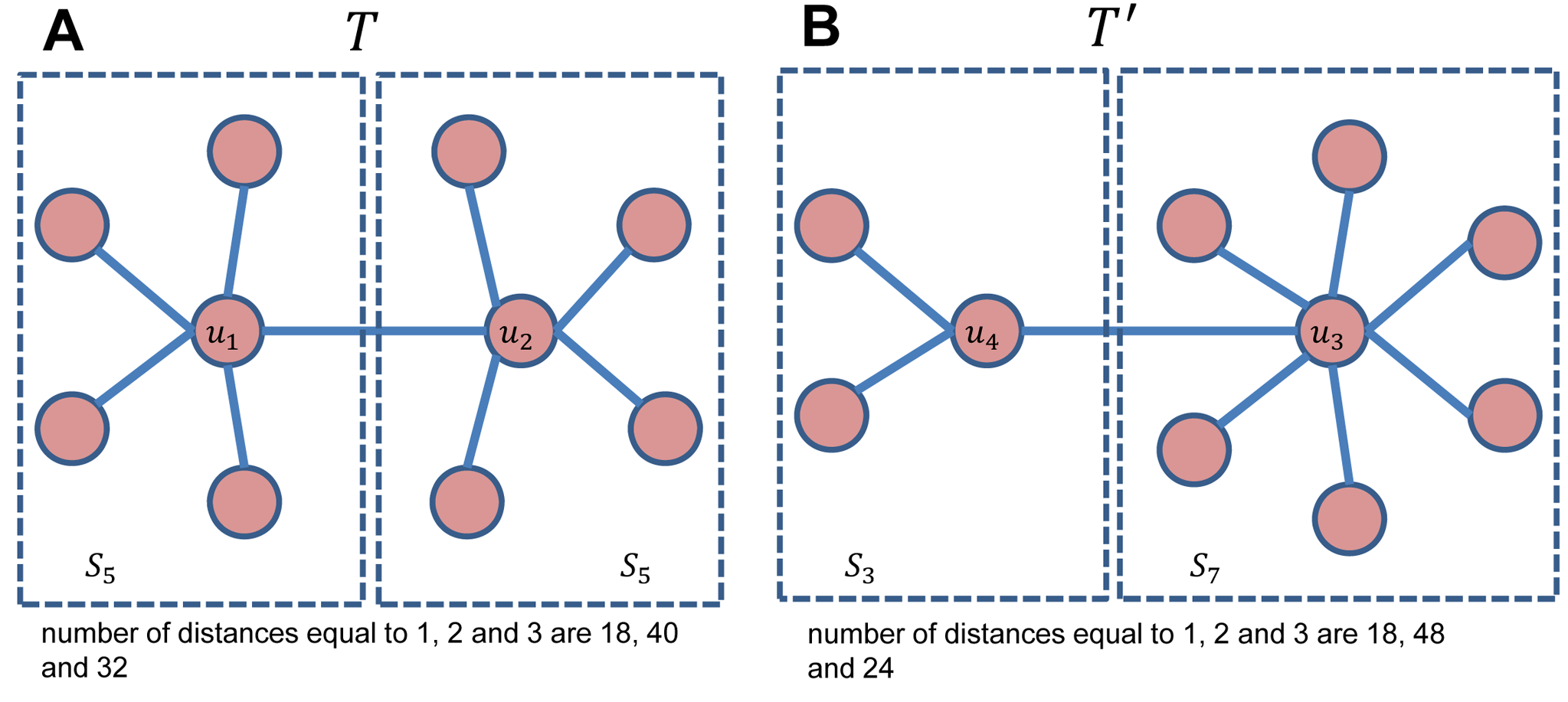

Supplement: Figure S2 — Illustration of Lemma 3. Here . From the counts of the distances above, it is clear that and . (TIF) [file pone.0078448.s002.tif]

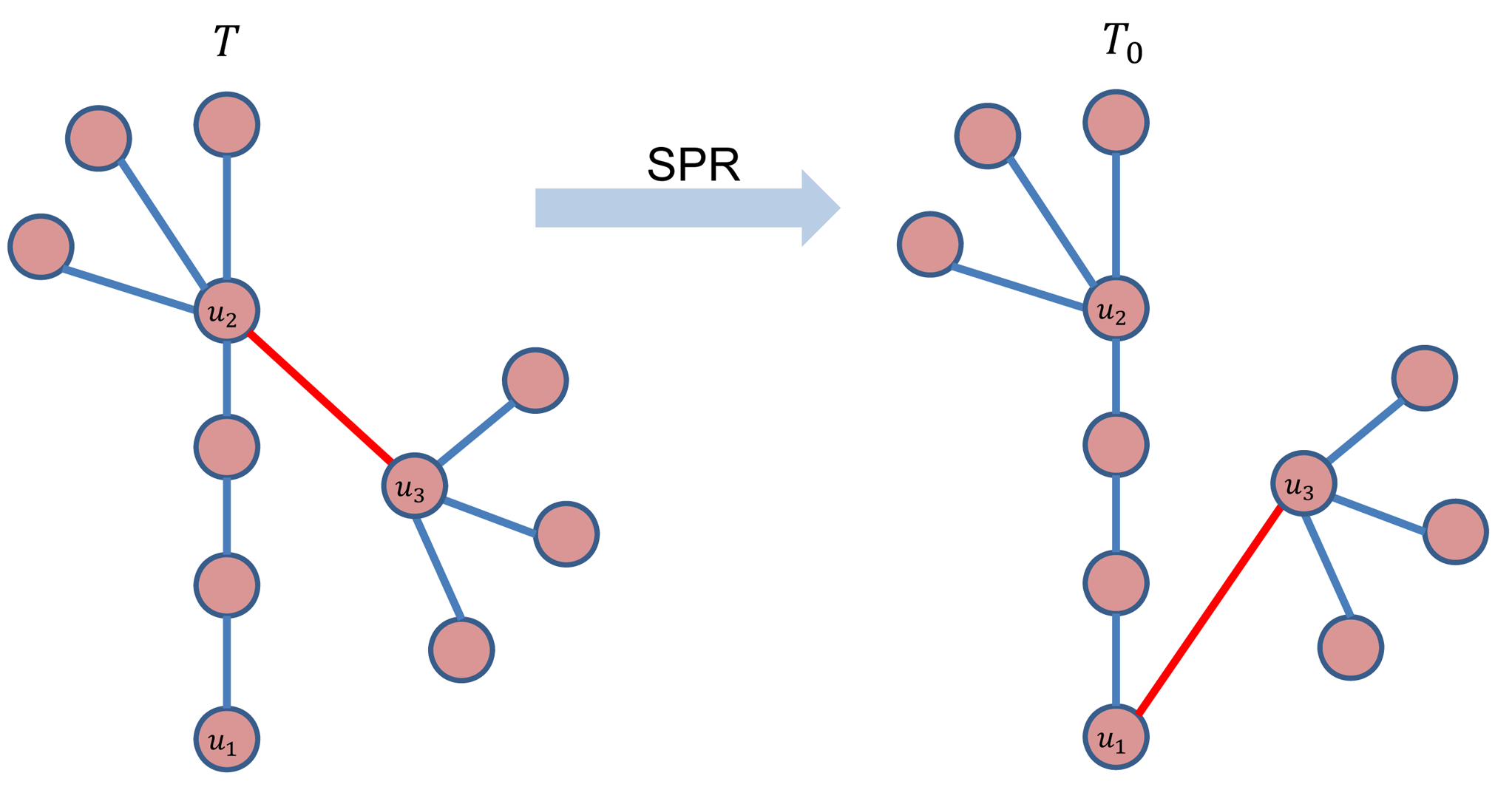

Supplement: Figure S3 — Illustration of the subtree pruning and regrafting algorithm. Here is obtained from first by deleting the edge and then connecting and . is proved to satisfy these properties: (i) ; (ii) ; and (iii) number of pendant nodes is one less than that of . (TIF) [file pone.0078448.s003.tif]
