# Supplementary material for: Sharp Bounds and Normalization of Wiener-Type Indices
Source: PLoS One. 2013 Nov 8;8(11):e78448. doi: 10.1371/journal.pone.0078448 (PMC3832646; doi:10.1371/journal.pone.0078448)
Supplement: Text S1 — Detailed proof for Theorems 1–4. (PDF) [file pone.0078448.s004.pdf]

Supporting information file for Sharp Bounds and Normalization of Wiener-type Indices

We describe here detailed proofs of Theorems 1-4. We start with some definitions and three Lemmas.

A matrix $A = (a_{ij})_{1 \le i,j \le n}$ is majorized by matrix $B = (b_{ij})_{1 \le i,j \le n}$, denoted by $A \preccurlyeq B$ or $B \succcurlyeq A$ if and only if

$$a_{(i)} \le b_{(i)} \quad \text{for } 1 \le i \le n \times n,$$

where $a_{(i)}$ and $b_{(i)}$ are the i-th smallest elements in A and B. A is strictly majorized by B, denoted by $A \prec B$ or $B \succ A$ if and only if

$$a_{(i)} \le b_{(i)} \quad \text{for } 1 \le i \le n \times n$$

and

$$a_{(i)} < b_{(i)} \quad \text{for some } i.$$

Matrices A and B are said to be equivalent, denoted by $A \equiv B$ if and only if

$$a_{(i)} = b_{(i)} \quad \text{for } 1 \le i \le n \times n.$$

Majorization, strict majorization, and equivalent between two vectors $A = (a_i)_{1 \le i \le n}$ and $B = (b_i)_{1 \le i \le n}$ are defined similarly.

Let G be a graph, define $V(G)$ as the set of nodes in G, and $E(G)$ as the set of edges in G. Let $\deg_G(u)$ denote the degree of node u in graph G. When there is no risk of ambiguity which graph G we are considering, we abbreviate $\deg_G(u)$ to $\deg(u)$. Define $ne(u) = \{v \in V(G) : (u, v) \in E(G)\}$ and call it neighborhood of node u. A node of degree 1 is called a pendant node or a leaf. A node which is not a pendant node is called an internal node.

A tree is called a starlike tree if it has exactly one node of degree greater than two. Figures 1(c), 1(f), and 1(g) show 8-node starlike trees with maximum degree equal to 5, 4, and 5 respectively.

Lemma 1 *Let T be a connected tree, u_1 a pendant node and u_2 an internal node. Suppose all nodes, if there is any, in the shortest path connecting u_1 and u_2 are of degree 2. Then*

$$(d(u_2, v))_{v \in V(T)} \quad \prec \quad (d(u_1, v))_{v \in V(T)}.$$

Proof. Let P_{u_1,u_2} denote the path connecting u_1 with u_2. For $v \in V(T) \backslash V(P_{u_1,u_2})$

$$\begin{aligned} d(u_1, v) &= d(u_1, u_2) + d(u_2, v) \\ &> d(u_2, v). \end{aligned}$$

And

$$d(u_1, v)_{v \in V(P_{u_1,u_2})} \equiv d(u_2, v)_{v \in V(P_{u_1,u_2})}.$$

Thus

$$(d(u_2, v))_{v \in V(T)} \prec (d(u_1, v))_{v \in V(T)}.$$

□

Lemma 2 *Consider two distinct trees T_1 and T_2. Let $u_1, u_2 \in V(T_1)$ with u_1 of degree at least 2 and u_2 a pendant node satisfying the property that any node, if there is any, on the shortest path connecting u_1 and u_2 is of degree 2. Let $u_3 \in V(T_2)$. A new tree T is constructed by connecting u_1 and u_3, and T' is constructed by connecting u_2 and u_3. Then,*

$$D(T) \prec D(T').$$

Proof. Observe that

$$(d(v_1, v_2))_{v_1, v_2 \in V(T_1)} \equiv (d'(v_1, v_2))_{v_1, v_2 \in V(T_1)},$$
$$(d(v_1, v_2))_{v_1, v_2 \in V(T_2)} \equiv (d'(v_1, v_2))_{v_1, v_2 \in V(T_2)}.$$

For $v_1 \in V(T_2)$, we have

$$\begin{aligned} & (d'(v_1, v_2))_{v_2 \in V(T_1)} \\ \equiv\ & d'(v_1, u_3) + 1 + (d'(u_2, v_2))_{v_2 \in V(T_1)} \\ \equiv\ & d(v_1, u_3) + 1 + (d(u_2, v_2))_{v_2 \in V(T_1)} \end{aligned}$$

and

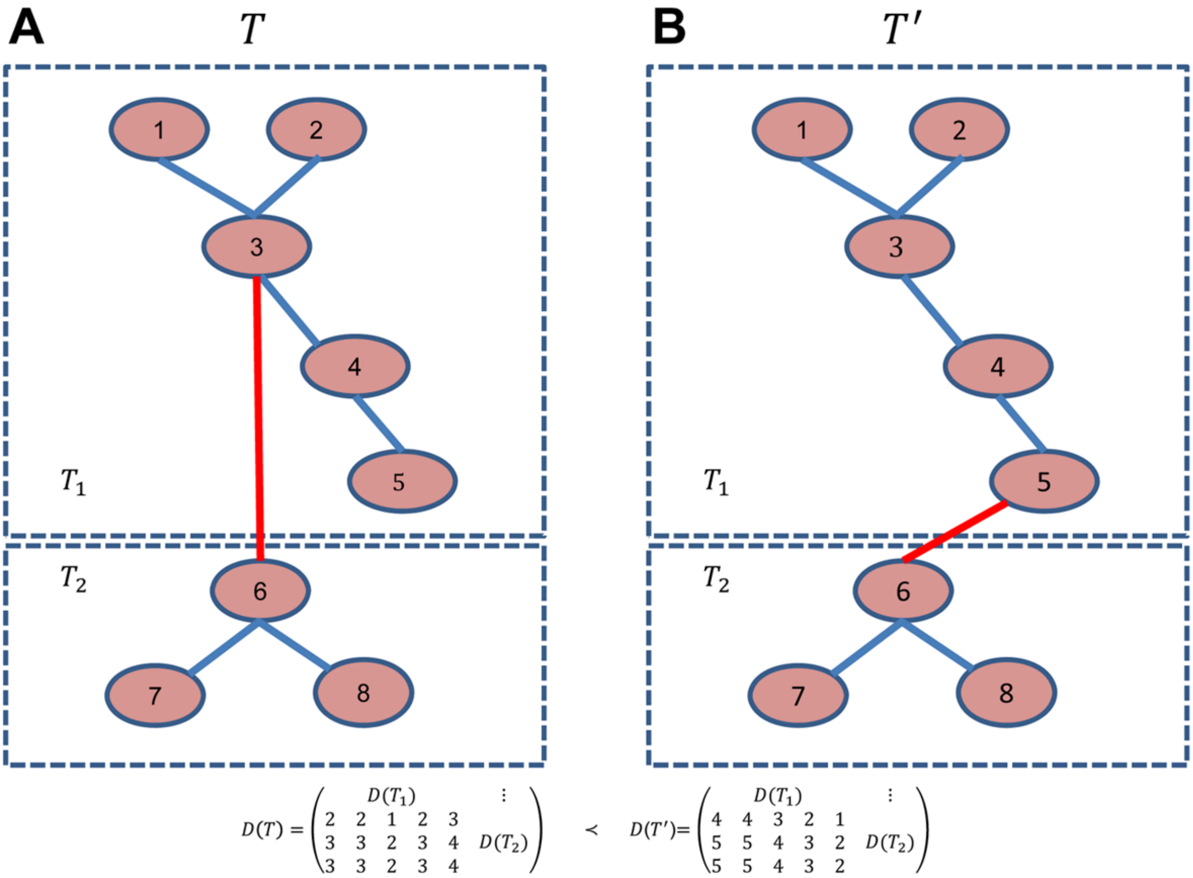

Figure S1. Illustrating the choices of u_1, u_2 and u_3 in Lemma 2. Here T_1 has 5 nodes, T_2 3 nodes. We choose $u_1 = 3, u_2 = 5$ and $u_3 = 6$. Tree T is constructed by joining u_1 and u_3 while T' by joining u_2 and u_3. $D(T)$ and $D(T')$ are 8×8 matrices where the first 5 columns correspondent to the 5 nodes in T_1, and the last 3 rows correspondent to the 3 nodes in T_2.

$$
\begin{aligned}
& (d(v_1, v_2))_{v_2 \in V(T_1)} \\
\equiv \quad & d(v_1, u_3) + 1 + (d(u_1, v_2))_{v_2 \in V(T_1)} \\
\prec \quad & (d'(v_1, v_2))_{v_2 \in V(T_1)}.
\end{aligned}
$$

Thus $D(T) \prec D(T')$. $\square$

Manipulations in Lemma 2 are illustrated in Figure S1.

Starting from a tree T with m number of nodes with maximum degree $\Delta(T)$. If $m \geq 2$, Lemma 2 can be iteratively applied to construct a tree T' such that the maximum degree is equal to that of T but the

number of nodes in T' with the maximum degree is reduced by 1. If $m = 1$, then Lemma 2 can also be iteratively applied to construct a tree T' with maximum degree $\Delta(T') = \Delta(T) - 1$.

Lemma 3 *Given $i + j = k + \ell = n$, $1 \leq \ell < i \leq j < k$, T is created by connecting internal node u_1 of S_i and internal node u_2 of S_j. T' is created by connecting internal node u_3 of S_k and internal node u_4 of S_ℓ. Then*

$$\begin{aligned}(d'(u_3, v))_{v \in V(T')} &\prec (d(u_1, v))_{v \in V(T)},\\ D(T') &\prec D(T).\end{aligned}$$

Proof. Note that $|V(T)| = |V(T')| = n$.

Note also that $(d(u_1, v))_{v \in V(T)}$ has 1 entry equals to 0, i entries equal to 1 and $j - 1$ entries equal to 2. Similarly $(d'(u_3, v))_{v \in V(T')}$ has 1 entry equals to 0, k entries equal to 1 and $\ell - 1$ entries equal to 2. Thus $(d(u_3, v))_{v \in V(T')} \prec (d'(u_1, v))_{v \in V(T)}$ proving the first majorization.

Both $D(T)$ and $D(T')$ have n entries equal to 0, $2(n - 1)$ entries equal to 1. $D(T)$ has $2(i - 1)(j - 1)$ entries equal to 3 and the rest of entries 2, $D(T')$ has $2(k - 1)(\ell - 1)$ entries equal to 3 and the rest of entries 2. Since $(k - 1)(l - 1) < (i - 1)(j - 1)$, thus $D(T') \prec D(T)$ proving the second majorization, and hence the proof of Lemma 3. □

Manipulations in Lemma 3 are illustrated in Figure S2, where $n = 10, i = j = 5, \ell = 3, k = 7$.

Proof of Theorem 2

In this section we will find upper and lower bounds of $W_f(T)$ for $T \in \mathcal{T}_n$. Lemmas 4 and 5 are dedicated to investigate the relationship between a tree's distance matrix and its maximum degree.

Consider the following subtree pruning and regrafting (SPR) algorithm:

Input $T \in \mathcal{T}_n$ with $\Delta(T) \geq 3$:

1. Choose a pendant node u_1, and an internal node u_2 with $\deg(u_2) \geq 3$ satisfying the condition that all nodes lying on the shortest path connecting u_1 and u_2, if any, are of degree 2.

2. Choose $u_3 \in ne(u_2)$ such that u_3 does not lie on the shortest path connecting u_1 and u_2.

3. A new tree $T^0 \in \mathcal{T}_n$ is constructed by first deleting (u_2, u_3) and then connecting u_3 to u_1.

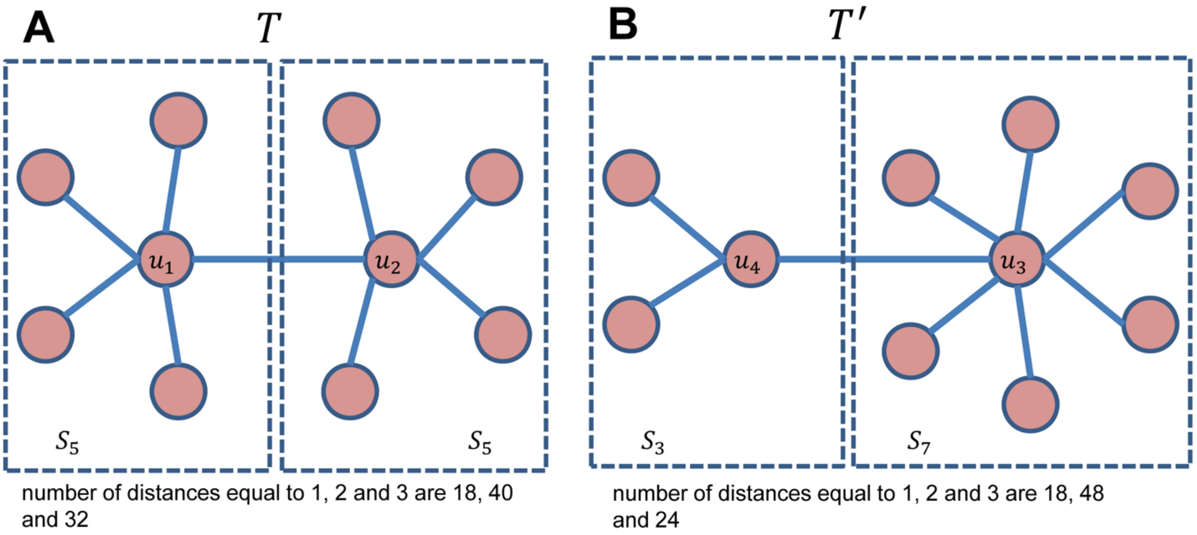

Figure S2. Illustration of Lemma 3. Here $n = 10, i = j = 5, \ell = 3, k = 7$. From the counts of the distances above, it is clear that $(d'(u_3, v))_{v \in V(T')} \prec (d(u_1, v))_{v \in V(T)}$ and $D(T') \prec D(T)$.

This algorithm outputs a tree T^0 with these properties: (i) $D(T) \prec D(T^0)$; (ii) $\Delta(T) - 1 \leq \Delta(T^0) \leq \Delta(T)$; and (iii) number of pendant nodes is one less than that of T.

To see this, let P_{u_1,u_2} denote the path connecting u_1 with u_2. Observe that

$$\begin{aligned} & (d(v_1, v_2))_{v_1, v_2 \in V(T) \setminus V(P_{u_1,u_2})} \\ \equiv \; & (d^0(v_1, v_2))_{v_1, v_2 \in V(T) \setminus V(P_{u_1,u_2})} \end{aligned}$$

and

$$\begin{aligned} & (d(v_1, v_2))_{v_1, v_2 \in V(P_{u_1,u_2})} \\ \equiv \; & (d^0(v_1, v_2))_{v_1, v_2 \in V(P_{u_1,u_2})}. \end{aligned}$$

For $v_1 \in V(T) \setminus V(P_{u_1,u_2})$, we have

$$\begin{aligned} & (d(v_1, v_2))_{v_2 \in P_{u_1,u_2}} \\ \equiv \; & d(v_1, u_3) + 1 + (d(u_2, v_2))_{v_2 \in P_{u_1,u_2}} \end{aligned}$$

and

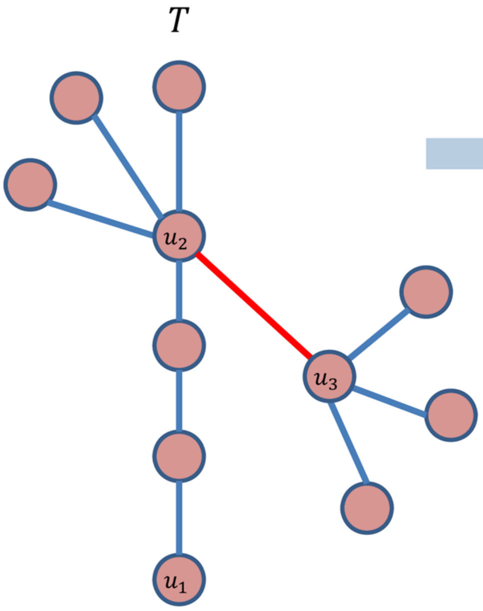

Figure S3. Illustration of the subtree pruning and regrafting algorithm. Here T_0 is obtained from T first by deleting the edge (u_2, u_3) and then connecting u_1 and u_3. T_0 is proved to satisfy these properties: (i) $D(T) \prec D(T^0)$; (ii) $\Delta(T) - 1 \leq \Delta(T^0) \leq \Delta(T)$; and (iii) number of pendant nodes is one less than that of T.

$$\begin{aligned}
& (d^0(v_1, v_2))_{v_2 \in P_{u_1,u_2}} \\
\equiv \quad & d^0(v_1, u_3) + 1 + (d^0(u_1, v_2))_{v_2 \in P_{u_1,u_2}} \\
\equiv \quad & d(v_1, u_3) + 1 + (d(u_1, v_2))_{v_2 \in P_{u_1,u_2}} \\
\succ \quad & (d(v_1, v_2))_{v_2 \in P_{u_1,u_2}} \quad \text{by Lemma 2.}
\end{aligned}$$

Thus $D(T) \prec D(T^0)$ and property (i) follows. Since $\deg_{T^0}(u_2) = \deg_T(u_2) - 1$, $\deg_{T^0}(u_1) = 2$, $\deg_{T^0}(u) = \deg_T(u)$ for $u \neq u_1, u_2$. Then properties (ii) and (iii) follow.

Manipulations of SPR algorithms are illustrated in Figure S3.

Lemma 4 *Let $T \in \mathcal{T}_n$ with $\Delta(T) \geq 3$. There exists $T' \in \mathcal{T}_n$ such that $\Delta(T') = \Delta(T) - 1$ and*

$$D(T) \quad \prec \quad D(T').$$

Proof. Let ℓ be the number of pendant nodes in T. Apply SPR algorithm to T to obtain T^0. If

$\Delta(T^0) = \Delta(T) - 1$, then we stop and take $T' = T^0$. Otherwise let $T = T^0$ and apply SPR algorithm again. We repeat this algorithm until we obtain the desired tree T'. Note that this algorithm will be repeated at most ℓ times to get the desired tree. $\square$

Lemma 5 *Let $T \in \mathcal{T}_n$ with $2 \leq \Delta(T) < n-1$. There exists $T' \in \mathcal{T}_n$ such that $\Delta(T') = \Delta(T) + 1$ and*

$$D(T') \prec D(T).$$

Proof. We write $\Delta(T) = k$. Choose $u \in V(T)$ with degree m in such a way that all its neighbors except one are pendant nodes. Write $ne(u) = \{u_1, \ldots, u_{m-1}, u_m\}$ where u_m is the only internal node in T. We consider two cases: 1: $m - 1 + \deg_T(u_m) < k+1$ and 2: $m - 1 + \deg_T(u_m) \geq k+1$.

1. A new tree T^0 is constructed by deleting edge (u, u_j), and then connecting u_j to u_m for $1 \leq j \leq m-1$. We claim that T^0 satisfies that $\Delta(T^0) = k$ and $D(T^0) \prec D(T)$. Since $\deg_{T^0}(v) = \deg_T(v), v \in V(T)\backslash\{u, u_m\}$, $\deg_{T^0}(u) = 1$, $\deg_{T^0}(u_m) = \deg_T(u_m) + m - 1 \leq k$, so $\Delta(T^0) = k$. $D(T^0) \prec D(T)$ follows from Lemma 3. Let $T = T^0$ and repeat this procedure again. Note that the number of pendant nodes in T increases by 1 for each application of this procedure.

2. A new tree T^0 is constructed by deleting edge (u, u_j), and connecting u_j to u_m for $1 \leq j \leq k - \deg_T(u_m) + 1$. As in case 1, T^0 satisfies $D(T^0) \prec D(T)$. Since $\deg_{T^0}(v) = \deg_T(v), v \in V(T)\backslash\{u_1, u_m\}$, $\deg_{T^0}(u) = \deg_T(u) - (k+1-\deg_T(u_m)) < k$, $\deg_{T^0}(u_m) = k+1$, so $\Delta(T^0) = k+1$. Let $T' = T^0$ and T' satisfies conditions in Lemma 5.

In either case, we shall eventually produce a tree as required in Lemma 5. $\square$

Since the star graph has the largest maximum degree, and the path graph has the smallest maximum degree among trees in $\mathcal{T}_n$, by Lemmas 4 and 5, we obtain the following corollary.

Corollary 1 *Let $T \in \mathcal{T}_n$ with $2 < \Delta(T) < n-1$. Then*

$$D(S_n) \prec D(T) \prec D(P_n).$$

Proof of Theorem 2 Applying Corollary 1 and the fact that f is increasing. $\square$

Proof of Theorem 1

Define $\mathcal{G}_n(m)$ as a set of connected graphs with the number of nodes n and the number of edges m.

First we will show that maximum value of $W_f(G)$ over $G \in \mathcal{G}_n(m)$ is a monotone function of the number of edges, m, of G.

Lemma 6 *Let $G \in \mathcal{G}_n$. Then $\max_{G\in\mathcal{G}_n(m)} W_f(G)$ and $\min_{G\in\mathcal{G}_n(m)} W_f(G)$ are decreasing functions in m.*

Proof. For any $G \in \mathcal{G}_n(m)$ with $D(G) = (d(i,j))_{1\le i,j\le n}$. Since $m \ge n$, G cannot be a tree and hence contains a cycle. Choose an edge in a cycle in G and delete it to form G'. Let's say the deleted edge is $(1,2)$. Note that $G' \in \mathcal{G}_n(m-1)$. Write $D(G') = (d'(i,j))_{1\le i,j\le n}$. Since $E(G') \subsetneqq E(G)$, $d(i,j) \le d'(i,j)$, $1 \le i < j \le n$, $W_f(G) \le W_f(G')$. So $\max_{G\in\mathcal{G}_n(m)} W_f(G) \le \max_{G\in\mathcal{G}_n(m-1)} W_f(G)$, for $m \ge n$.

Consider $n \le m \le \frac{n(n-1)}{2}$. For any $G \in \mathcal{G}_n(m-1)$, we connect two nodes with distance greater than 1 in G and call the resulting graph G''. Now $G'' \in \mathcal{G}_n(m)$ with $D(G'') = (d''(i,j))_{1\le i\le j\le n}$. Since $E(G) \subset E(G'')$, $d''(i,j) \le d(i,j)$, $1 \le i < j \le n$, thus $W_f(G'') \le W_f(G)$. So $\min_{G\in\mathcal{G}_n(k)} W_f(G) \le \min_{G\in\mathcal{G}_n(m-1)} W_f(G)$ for $m \ge n$. □

Proof of Theorem 1 From Lemma 6 we have

$$W_f(K_n) \le W_f(G) \le \max\{W_f(T) : T \in \mathcal{T}_n\}.$$

From Theorem 2

$$W_f(P_n) = \max\{W_f(T) : T \in \mathcal{T}_n\}.$$

Thus Theorem 1 follows. □

Proof of Theorem 3

In this section, we consider trees with a given maximum degree. The relationship between the distance matrix and the number of nodes with degree equal to maximum degree is investigated.

Lemma 7 *Let $T \in \mathcal{T}_n$ with n_1 nodes with degree equal to $\Delta(T)$. Suppose $n_1 \ge 2$ and $\Delta(T) \ge 3$. There exists $T' \in \mathcal{T}_n$ with $\Delta(T') = \Delta(T)$ and $n_1 - 1$ nodes with degree equal to $\Delta(T)$. Moreover, we have*

$$D(T) \prec D(T').$$

Proof. Let ℓ be the number of pendant nodes in T. Apply SPR algorithm to T to obtain T^0. If T^0 has $n_1 - 1$ nodes with degree equal to $\Delta(T)$, then we stop and take $T' = T^0$. Otherwise let $T = T^0$ and apply SPR algorithm again. We repeat this algorithm until we obtain desired tree T'. Note that this algorithm will be repeated at most $\ell - n_1 + 1$ times to obtain desired tree. $\square$

Corollary 2 *Let $T \in \mathcal{T}_n$ with $2 < \Delta(T) < n-1$. There exists a starlike tree T' with $\Delta(T) = \Delta(T')$ such that*

$$D(T) \prec D(T').$$

Corollary 2 states that among trees with equal maximum degree, distance matrix of a tree with more than one node with maximum degree is strictly majorized by a distance matrix of a starlike tree. Next to find a tree whose distance matrix majorizes all starlike trees.

Lemma 8 *Let T be a starlike tree with $\Delta(T) = k \geq 3$. Then*

$$D(T) \quad \preceq \quad D(B_{n,k+1}),$$

with equality holds if and only if T is $B_{n,k+1}$.

Proof. Assume T is non-isomorphic to $B_{n,k+1}$. Denote by u the node with maximum degree k, by $u_1, \ldots, u_k$ pendant nodes in T, and by V_i set of nodes in the shortest path connecting node u and u_i, $1 \leq i \leq k$. Next a new tree T^0 is constructed by deleting edge $(u_{k-1}, ne(u_{k-1}))$ and connecting u_{k-1} to u_k.

For $i, j \in V \backslash \{u_{k-1}\}$,

$$d(i,j) \quad = \quad d^0(i,j).$$

For $i \in V \backslash (V_{k-1} \cup V_k)$

$$\begin{aligned} d(i, u_{k-1}) &= d(i,u) + d(u, u_{k-1}) \\ d^0(i, u_{k-1}) &= d^0(i,u) + d^0(u, u_{k-1}) \\ &= d(i,u) + d(u, u_k) + 1 \end{aligned}$$

thus

$$d(i, u_{k-1}) \quad < \quad d^0(i, u_{k-1}).$$

And

$$(d(i, u_{k-1}))_{V_{k-1} \cup V_k} \quad \equiv \quad (d^0(i, u_{k-1}))_{V_{k-1} \cup V_k},$$

since both vectors are distances of a pendant node to other nodes in one path with length $d(u_{k-1}, u_k)$. Thus $D(T) \prec D(T^0)$. If T^0 satisfies $d^0(u, u_1) = \cdots = d^0(u, u_{k-1}) = 1$, then we stop and T^0 is $B_{n,k+1}$. Otherwise let $T = T^0$ and we repeat this process until get tree $B_{n,k+1}$. Note that this algorithm will be repeated $n - k - d(u, u_k)$ times. □

Lemma 9 *For $k \geq 3$,*

$$D(B_{n,k+1}) \quad \prec \quad D(B_{n,k})$$

Proof. Lemma 9 follows directly from Lemmas 4 and 8. □

Proof of Theorem 3 Applying Lemma 8 and the fact that f is increasing. □

Remark It has been proven in corollary 3.5 of [1] that

$$W_f(T_n(k)) = \min\{W_f(T) : T \in \mathcal{T}_n, \quad \Delta(T) = k\} \qquad (\star)$$

where $T_n(k)$ is a k-ary tree, also called Volkmann tree [2]. It remains open whether

$$D(T_n(k)) \preceq D(T) \quad \text{ for } T \in \mathcal{T}_n,\ \Delta(T) = k \qquad (\star\star)$$

holds for all k, n and $k \leq n$. We have verified that $(\star\star)$ holds for $6 \leq n \leq 9$ and $k = 3$. If $(\star\star)$ is true for all n and k, it provides an alternative proof of

$$W_f(T_n(k)) \leq W_f(T)$$

for $T \in \mathcal{T}_n$, $\Delta(T) = k$, and f monotonically increasing.

Proof of Theorem 4

Proof. Let T be a spanning tree of G satisfying $\Delta(T) = k$. Similar to the proof of Theorem 1, one can prove that $D(G) \preceq D(T)$. By Theorem 3, $D(T) \preceq D(B_{n,k+1})$. Thus $W_f(G) \leq W_f(B_{n,k+1})$. □

References

1. Schmuck NS, Wagner SG, Wang H (2012) Greedy trees, caterpillars, and wiener-type graph invariants. Match-Communications in Mathematical and Computer Chemistry 68: 273.

2. Fischermann M, Hoffmann A, Rautenbach D, Székely L, Volkmann L (2002) Wiener index versus maximum degree in trees. Discrete Applied Mathematics 122: 127–137.
