# Supplementary material for: Correction: Sharp Bounds and Normalization of Wiener-Type Indices
Source: PLoS One. 2014 Apr 15;9(4):e95652. doi: 10.1371/journal.pone.0095652 (PMC3988199; doi:10.1371/journal.pone.0095652)
Supplement: File S1 — Originally published, uncorrected article. [file pone.0095652.s001.pdf]

Sharp Bounds and Normalization of Wiener-Type Indices

Dechao Tian[1], Kwok Pui Choi[1,2]*

1 Department of Statistics and Applied Probability, National University of Singapore, Singapore, Singapore, 2 Department of Mathematics, National University of Singapore, Singapore, Singapore

Abstract

Complex networks abound in physical, biological and social sciences. Quantifying a network's topological structure facilitates network exploration and analysis, and network comparison, clustering and classification. A number of Wiener type indices have recently been incorporated as distance-based descriptors of complex networks, such as the R package QuACN. Wiener type indices are known to depend both on the network's number of nodes and topology. To apply these indices to measure similarity of networks of different numbers of nodes, normalization of these indices is needed to correct the effect of the number of nodes in a network. This paper aims to fill this gap. Moreover, we introduce an f-Wiener index of network G, denoted by $W_f(G)$. This notion generalizes the Wiener index to a very wide class of Wiener type indices including all known Wiener type indices. We identify the maximum and minimum of $W_f(G)$ over a set of networks with n nodes. We then introduce our normalized-version of f-Wiener index. The normalized f-Wiener indices were demonstrated, in a number of experiments, to improve significantly the hierarchical clustering over the non-normalized counterparts.

Citation: Tian D, Choi KP (2013) Sharp Bounds and Normalization of Wiener-Type Indices. PLoS ONE 8(11): e78448. doi:10.1371/journal.pone.0078448

Editor: Fabio Rapallo, University of East Piedmont, Italy

Received July 3, 2013; **Accepted** September 11, 2013; **Published** November 8, 2013

Copyright: © 2013 Tian, Choi. This is an open-access article distributed under the terms of the Creative Commons Attribution License, which permits unrestricted use, distribution, and reproduction in any medium, provided the original author and source are credited.

Funding: This work was supported by funds provided by Ministry of Education (R146-000-134-11). The funders had no role in study design, data collection and analysis, decision to publish, or preparation of the manuscript.

Competing Interests: The authors have declared that no competing interests exist.

* E-mail: stackp@nus.edu.sg

Introduction

Recent years witness exponential growth of available biological network data. Thanks to past decades' breakthrough in biotechnology, researchers now are able to interrogate molecular interactions at systems level. It has since been observed that topological properties of these networks provide important insight into the functions of proteins, and their relationship with one another [1–8]. For examples, degree distribution, average clustering coefficient, diameter, centrality, lethality and graphlet distribution have been extensively studied. Hopefully, based on a carefully chosen list of network topological properties and methods in quantifying them, a complex network is adequately summarized in the form of a numerical d-dimensional vector where d is the number of topological properties in consideration. This representation enables us to take full advantage of a host of classification and clustering techniques to compare complex networks.

A significant step towards this direction is facilitated by the introduction of the R package QuACN by Mueller et al. [9]. QuACN computes the values of different categories of descriptors in a network. One such category is the distance-based descriptors which include Wiener index, Harary index, etc. The use of Wiener index and related type of indices dates back to the seminal work of Wiener in 1947 [10,11]. Wiener introduced his celebrated index to predict the physical properties, such as boiling point, heats of isomerization and differences in heats of vaporization, of isomers of paraffin by their chemical structures. Viewing the chemical structure of an isomer as a connected graph, the Wiener index is defined as $\sum_{i,j} d(i,j)$ where i,j represent nodes in the graph, $d(i,j)$ the distance between nodes i and j which is defined as the length of a shortest path between them, and the sum is over all pairs of nodes in the graph. Wiener index has since inspired many distance-based descriptors in Chemometrics. These include Harary index [12], hyper Wiener index [13], q-analog of Wiener index [14], Wiener polynomial [15], Q-index [16], Balaban J index [17], and information indices [18–20]. These indices, or commonly called descriptors, play significant roles in quantitative structure-activity relationship/quantitative structure-property relationship (QSAR/QSPR) models [21].

It is known that the Wiener type indices depend both on a network's number of nodes and its topology. When the numbers of nodes in the networks are equal, as in the applications to isomers, these indices provide informative measures of the branching property of the networks and hence a fair comparison among them. However, when they are used to measure similarities of networks with different numbers of nodes, the intended measure of topological structures will be masked by the sizes of the networks. Normalization of a Wiener type index expectedly minimizes the effect of the network's number of nodes and hence brings forth its topological structure better. Furthermore, it is also desirable for the normalized index to take value in an absolute scale for better understanding and interpretation. This paper seeks to fill this gap. The normalization introduced in definition 2 below fulfils this purpose. This definition will be of limited practical value if the sharp upper and lower bounds of the index on a graph cannot be found explicitly. The objective of this article is three-fold. First, introduce a very general Wiener type index. We call it f-Wiener index, and denote it by $W_f(G)$ for a graph G. This definition includes all known Wiener type indices as special cases. Second, identify the maximum and minimum values of $W_f(G)$ over a class of connected networks G or a class of connected trees G. We are able to derive explicit formulas for these optimal values. Third, propose a normalized version, $W_f^*(G)$ which takes value in [0,1] for better interpretation and network comparison.

This paper is organized as follows. We first introduce some standard graph-theoretic notations and recall some special graphs. We then introduce the functional analog of Wiener index, $W_f(G)$, and our proposed normalized versions of this functional Wiener index in the method section. In the result section, we provide our main results Theorems 1 to 4. Theorem 1 gives the maximum and the minimum of $W_f(G)$ over the set of connected graphs of n nodes, and characterization of graphs achieving the maximum or the minimum. Theorem 2 gives a parallel result when the maximum and minimum are taken over the set of connected trees of n nodes. Theorem 3, (respectively Theorem 4) identifies the maximum of $W_f(G)$ over the set of connected graphs (respectively connected trees) of n nodes with specified maximum degree. We also give a brief description of related works in next section. Then, we consider special cases of f in $W_f(G)$ to provide explicit expressions of the maximum and the minimum of Wiener, Harary, hyper Wiener, generalized Wiener indices. In the experiment section, we report the performance of hierarchical clustering based on the usual Wiener type indices and the normalized version of these in our experiments. We end with conclusions section of this paper.

Methods

Definitions and Terminologies

Let $G=(V,E)$ be a simple (that is, no self-loops nor multiple edges) connected graph on n nodes where $V=\{1,\ldots,n\}$ and $E\subseteq V\times V$. Denote by $N(G)$ as the number of nodes in G. Let $\mathcal{G}_n$ denote the set of all simple, connected graphs with n nodes. A graph having no cycles is called a tree, and we let $\mathcal{T}_n$ denote the set of all connected trees with n nodes. The distance $d(i,j)$ between any pair of nodes, i and j, in G is the number of edges in a shortest path from i to j. Let $D(G)=[d(i,j)]_{1\le i,j\le n}$ be the distance matrix. We denote the maximum degree of G by $\Delta(G)$.

Figure 1 shows some special graphs we frequently refer to in this paper. A path graph, P_n, is a graph that can be drawn so that all of its vertices and edges lie on a straight line. Figure 1(a) shows P_8. A star, S_n, is a tree with one internal node and $n-1$ leaves. S_8 is shown in Figure 1(b). A complete graph, K_n, is a graph with n nodes in which every pair of distinct nodes is connected by an edge. A caterpillar, $C_{n,k}$, is a tree with a central path with number of nodes $\in[n/(k+1),(n+k)/(k+1)]$ where at most one end node of the central path has less than k leaves, each of the other nodes in the central path has k leaves. Figures 1(d) and 1(e) show caterpillars $C_{12,2}$ and $C_{8,3}$ respectively. A broom $B_{n,k}$ is a tree joining a star S_{k+1} and a path P_{n-k-1} by attaching a pendant node (or leaf) in P_{n-k-1} to a pendant node of S_{k+1}. For examples, brooms $B_{8,4}$ and $B_{8,5}$ are shown in Figures 1(f) and 1(g) respectively. A kite $K_{n,\ell}$ is a graph obtained from connecting two end nodes one from a complete graph K_ℓ and one from a path $P_{n-\ell}$. Figure 1(h) shows a kite $K_{8,4}$.

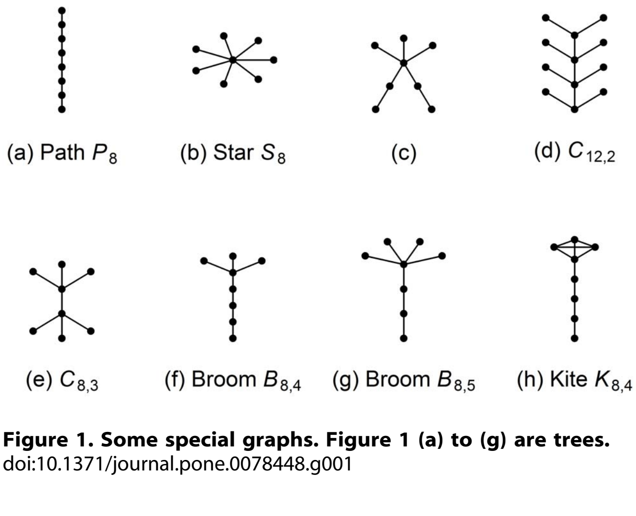

Figure 1. Some special graphs. Figure 1 (a) to (g) are trees.
doi:10.1371/journal.pone.0078448.g001

Throughout this paper, f denotes a monotone function defined on nonnegative integers. We define a functional-analog Wiener index below. Our definition contains the Wiener index, Harary index, hyper Wiener index, compactness, average efficiency, generalized Wiener index, Wiener polynomial, Q-index, q-analogy of Wiener index as special cases. For detail, see subsection Important special cases. We abbreviate it as f-Wiener index. Thanks to an anonymous reviewer of this article, this definition has also been independently introduced by Schmuck et al. [22].

Definition 1. *The f-Wiener index of $G\in\mathcal{G}_n$ is defined by*

$$W_f(G)=\sum_{1\le i<j\le n} f(d(i,j)).$$

Here $d(i,j)$ denotes the shortest distance between nodes i and j.

The number of nodes of G has a very strong effect on Wiener type indices (see Results section). In order to apply f-Wiener index for comparing networks, which often differ in the numbers of nodes, we are led to propose a normalized version for graphs and a normalized version for trees for better interpretation of the index.

Definition 2. *(a) The normalized f-Wiener index for a graph $G\in\mathcal{G}_n$ is defined as*

$$W_f^*(G)=\frac{M_f-W_f(G)}{M_f-m_f}.$$

Here $M_f=\max_{H\in\mathcal{G}_n}\{W_f(H)\}$ and $m_f=\min_{H\in\mathcal{G}_n}\{W_f(H)\}$.

(b) The normalized f-Wiener index for a tree $T\in\mathcal{T}_n$ is similarly defined where the maximum M_f and the minimum m_f are taken over $\mathcal{T}_n$ instead.

These normalized versions will be of limited practical value if one cannot compute M_f nor m_f. Our main results, stated in Theorems 1 and 2, show that these optimal upper and lower bounds can be easily computed. Moreover, they characterize those graphs which attain the maximum or the minimum.

By definition, $W_f^*(G)$ takes values in [0,1]. When f is a non-decreasing function, Theorem 1 below shows that $W_f^*(G)=0$ if and only if G is a path graph, and $W_f^*(G)=1$ if and only if G is a complete graph. So $W_f^*(G)\approx 0$ (respectively, $W_f^*(G)\approx 1$) suggests G looks like a path graph (respectively, a complete graph). And hence the numerical value of $W_f^*(G)$ provides an indication how G is like.

Effect of Number of Nodes on Wiener Type Indices

It is known that the Wiener index for a connected graph with n nodes ranges from $n(n-1)/2$ to $n(n-1)(n+1)/6$ (see Corollary 5 below or [23–25]). This wide range can be undesirable if it is used for comparing similarity of graphs with different number of nodes. For example, consider two path graphs, P_4 and P_5, with 4 nodes and 5 nodes respectively, and a star graph with 5 nodes, S_5. Values of the Wiener index for P_4,P_5 and S_5 are respectively 10, 20 and 16, giving the false impression that P_5 and S_5 are more similar than that of P_4 and P_5. However, values of the normalized Wiener index are 0 for P_4 and P_5, and 1 for S_5. This example is far from

being an isolated case, it can be shown that if the number of nodes of a path graph is at least 26% more than the number of nodes in another path graph, there exists a star graph whose Wiener index is closer to that of the path graph with smaller number of nodes.

The normalized Wiener index of S_n, star with n nodes, is $1-3/n$, suggesting stars of sufficiently large n, based on the normalized Wiener index, S_n is very similar to a complete graph. This is concordant with the fact that a K_n is the line graph of S_{n+1} [26].

Main Idea

A key ingredient in our proofs is a matrix majorization (see Supporting information file Text S1 for definition) argument. Given a connected graph G, we can transform it to another graph G' such that the distance matrix of G, $D(G)=[d(i,j)]_{1\leq i,j\leq n}$ majorizes the corresponding distance matrix of G'. Since Wiener index of G, or its generalization f-Wiener index for increasing function f, is the sum of the upper diagonal entries in the distance matrix, it follows that $W_f(G)\geq W_f(G')$. The construction of G' is fairly straightforward as can be seen in the proofs. The construction of G'' such that $D(G)$ is majorized by $D(G'')$ requires delicate and judicious pruning and regrafting. However, the essential idea remains the same. Technical details of proofs are given in supporting information file Text S1.

Results

We provide explicit expressions for the maximum and minimum of $W_f(G)$ over $\mathcal{G}_n$, and over $\mathcal{T}_n$ in Theorems 1 and 2 below. We also characterize those graphs or trees attaining the extremum. Theorems 3 and 4 concern trees or graphs with a specified maximum degree. For simplicity of presentations, we shall only state our results for non-decreasing function f. Analogous results for non-increasing f can be deduced easily by replacing f by $-f$.

Theorem 1 *Let f be a non-decreasing function on nonnegative integers, and $G\in\mathcal{G}_n$, then*

$$\frac{n(n-1)}{2}f(1)\leq W_f(G)\leq\sum_{i=1}^{n-1}(n-i)f(i).$$

The lower bound is attained if and only if G is K_n. The upper bound is attained if and only if G is P_n.

Theorem 2 *Let f be a non-decreasing function on nonnegative integers, and $T\in\mathcal{T}_n$, then*

$$\frac{(n-1)((n-2)f(2)+2f(1))}{2}\leq W_f(T)\leq\sum_{i=1}^{n-1}(n-i)f(i).$$

The lower bound is attained if and only if T is S_n. The upper bound is attained if and only if T is P_n.

Theorem 3 *Let f be a non-decreasing function on nonnegative integers. Then, for any $T\in\mathcal{T}_n$ with $\Delta(T)=k$, we have*

$$W_f(T)\leq W_f(B_{n,k+1}).$$

The upper bound is attained if and only if T is a broom $B_{n,k+1}$.

Theorem 4 *Let f be a non-decreasing function on nonnegative integers. Then, for any $G\in\mathcal{G}_n$ with $\Delta(G)=k$, we have*

$$W_f(G)\leq W_f(B_{n,k+1}).$$

Moreover,

$$W_f(B_{n,k+1})=\sum_{j=1}^{n-k+1}(n-j)f(j)+\frac{(k-1)(k-2)}{2}f(2).$$

Equality holds if and only if G is $B_{n,k+1}$.

Related Work

The proofs of Theorems 1 to 4 will be given in supporting information file Text S1. Theorem 2 has also been independently obtained by Wagner et al. (see Theorem 2.7 and Corollary 4.1 in [27]). Special cases of Theorems 1 to 4 for particular Wiener type index are known in the literature. For examples, the complete graph (respectively, the path graph) is shown to be the minimizer (respectively, maximizer) of the Wiener index among simple connected graphs with the same number of nodes in [23–25]. Similar conclusions are proved to hold for the hyper Wiener index in [25], and the Harary index in [28]. The results in Theorems 1 to 4 in its full generality as f-Wiener index are novel to the best knowledge of the authors. Moreover, we have provided a unifying methodology for the proofs.

Important Special Cases

Since its introduction, Wiener index has inspired many variants and thoroughly studied in a sizeable literature [29]. By choosing appropriate functions f, the f-Wiener index can be reduced to a number of commonly used descriptors as follows.

If we take $f(k)=k$, $W_f(G)$ written as $W(G)$ is the well-studied descriptor introduced by Wiener in 1947 [10,11].

Taking $f(k)=1/k$, the f-Wiener index is the Harary index [12], denoted by $H(G)$ which is shown to be more discriminating than the Wiener index [12]. Latora and Marchiori in 2001 [30], used a scaled version of the Harary index (more precisely, $f(k)=\frac{2}{n(n-1)k}$) to measure a network's efficiency in information exchange.

Taking $f(k)=k^\alpha$, where α can be positive or negative, the f-Wiener index is called generalized Wiener index, denoted by $W_\alpha(G)$ [31].

If $f(k)=(k^2+k)/2$, the f-Wiener index is known as the hyper Wiener index [13], denoted by $WW(G)$.

Taking $f(k)=\lambda^k$, where λ is regarded as a parameter, the f-Wiener index is called the Hosoya polynomial or Wiener polynomial [15]. With an additional factor 2, the Hosoya polynomial is called Q-index and denoted by $Q(\lambda)$ in [16].

The q-analog of the Wiener index, introduced by Zhang et al. (2012) in [14] is simply the f-Wiener index by choosing $f(k)=(1-q^k)/(1-q)=\sum_{t=0}^{k-1}q^t$.

Applications

By specializing f to various forms in Theorems 1 and 2, we provide below explicit sharp upper bounds and sharp lower bounds for the Wiener index $W(G)$, the Harary index $H(G)$, the

Table 1. Adjusted Rand Index (ARI) for clustering (or classification) of networks in our three experiments.

	Non-normalized	Normalized
Experiment 1.1	0.44 (0.02)	0.88 (0.07)
Experiment 1.2	0.41 (0.06)	1.00 (0.01)
Experiment 1.3	0.38 (0.10)	1.00 (0.00)
Experiment 1.4	0.36 (0.11)	0.97 (0.10)
Experiment 1.5	0.30 (0.12)	0.62 (0.07)
Experiment 2	0.10	1.00
Experiment 3	0.04	0.86

For experiments 1.1 to 1.5, we report the mean and the standard deviation (number in parenthesis) of ARI. Mean and standard deviation of ARI for experiments 1.1 to 1.5 under random clustering are 0 and 0.05 respectively.
doi:10.1371/journal.pone.0078448.t001

hyper Wiener index $WW(G)$, and the generalized Wiener index $W_\alpha(G)$ for $\alpha > 0$ and $\alpha < 0$.

Corollary 5 *Let G be a simple, connected graph with n nodes (that is, $G \in \mathcal{G}_n$), we have*

$$\frac{n(n-1)}{2} \le W(G) \le \frac{n(n-1)(n+1)}{6},$$

$$n\sum_{i=2}^{n-1}\frac{1}{i} + 1 \le H(G) \le \frac{n(n-1)}{2},$$

$$\frac{n(n-1)}{2} \le WW(G) \le \frac{n(n-1)(n+1)(n+2)}{24},$$

when $\alpha < 0$,

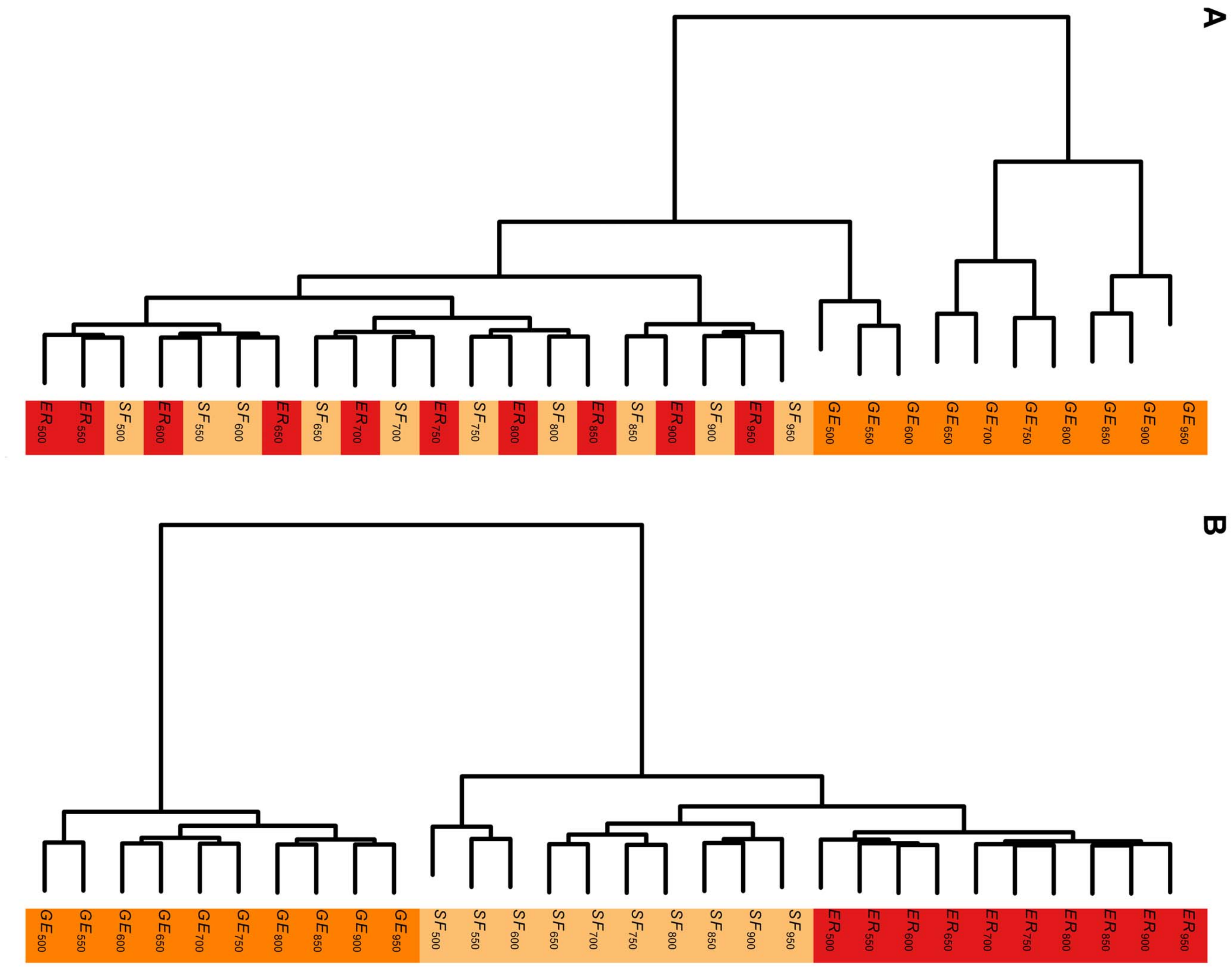

Figure 2. Hierarchical clustering of random networks. 30 networks with 10 each generated by the Erdos-Renyi (ER), scale-free (SF) and geometric (GE) random network models. Panel (A) shows the hierarchical clustering based on the f-Wiener indices (see Step 1 on page 8 for functions used). The adjusted rand index (ARI) for this clustering is 0.24. Panel (B) is the hierarchical clustering based on the normalized versions of the same f-Wiener indices. The ARI of this clustering is 0.67. Number of nodes chosen are 500, 550, …, 950, and p is 0.05 in the Erdos-Renyi model. A scale-free network with 500 nodes is denoted by SF_{500}. The others are denoted in a similar way.
doi:10.1371/journal.pone.0078448.g002

$$n\sum_{i=1}^{n-1} i^{\alpha} - \sum_{i=1}^{n-1} i^{\alpha+1} \le W_{\alpha}(G) \le \frac{n(n-1)}{2},$$

when $\alpha>0$,

$$\frac{n(n-1)}{2} \le W_{\alpha}(G) \le n\sum_{i=1}^{n-1} i^{\alpha} - \sum_{i=1}^{n-1} i^{\alpha+1}.$$

Corollary 6 *Let T be a tree with n nodes (that is, $T \in \mathcal{T}_n$), we have*

$$(n-1)^2 \le W(T) \le \frac{n(n-1)(n+1)}{6},$$

$$n\sum_{i=2}^{n-1}\frac{1}{i} + 1 \le H(T) \le \frac{(n-1)(n+2)}{4},$$

$$\frac{(n-1)(3n-4)}{2} \le WW(T) \le \frac{n(n-1)(n+1)(n+2)}{24},$$

when $\alpha<0$,

$$n\sum_{i=1}^{n-1} i^{\alpha} - \sum_{i=1}^{n-1} i^{\alpha+1} \le W_{\alpha}(T) \le ((n-2)2^{\alpha-1}+1)(n-1),$$

when $\alpha>0$,

$$((n-2)2^{\alpha-1}+1)(n-1) \le W_{\alpha}(T) \le n\sum_{i=1}^{n-1} i^{\alpha} - \sum_{i=1}^{n-1} i^{\alpha+1}.$$

Experiments

We describe below three experiments to compare the hierarchical clustering using normalized f-Wiener indices with the hierarchical clustering using non-normalized f-Wiener indices. Each experiments consists of 3 main steps.

Step 1: A collection of networks (or graphs) or trees, $\mathcal{C}$, are chosen to be clustered. The collection is detailed in each experiment below.

Step 2: Seven functions are chosen to form the f-Wiener indices. In all our experiments, we choose

$$f_1(k)=\sqrt{k}, f_2(k)=k, f_3(k)=\frac{k+k^2}{2},$$

and

$$f_4(k)=\frac{4k}{N(G)(N(G)-1)},$$

$$f_5(k)=k^{-1/2}, f_6(k)=k^{-1}, f_7(k)=k^{-2}.$$

The first four functions chosen are increasing and the f-Wiener indices correspond to the usual $W_{1/2}$ index, Wiener index, the hyper Wiener index and the compactness index. The remaining 3 functions chosen are decreasing and correspond to the $W_{-1/2}$ index, the Harary index and the W_{-2} index. Hopefully these indices collectively capture some essential characters of networks and useful for clustering. For $G \in \mathcal{C}$, we construct two characteristic vectors,

$$v_G = (W_{f_1}(G), \ldots, W_{f_7}(G)),$$

$$v_G^{\star} = (W_{f_1}^{\star}(G), \ldots, W_{f_7}^{\star}(G)).$$

Step 3: We adopt a clustering algorithm to cluster $\mathcal{C}$ using v_G and then produce a dendrogram. We do the same using v_G^{*}. Minimum variance method algorithm due to Ward [32] which is made available in R base package [33], was used in all the experiments. The computed the Adjusted Rand Index (ARI) in all the experiments are summarized in Table 1 below.

Experiment 1: Hierarchical Clustering of Random Networks

The collection of networks chosen for this experiment is the networks generated by some commonly used random network models, namely, Erdos-Renyi (ER) model [34,35], scale-free (SF) network model [36] and 3-D geometric model (GE) [37]. Each of these random network models is applied to generate 10 random

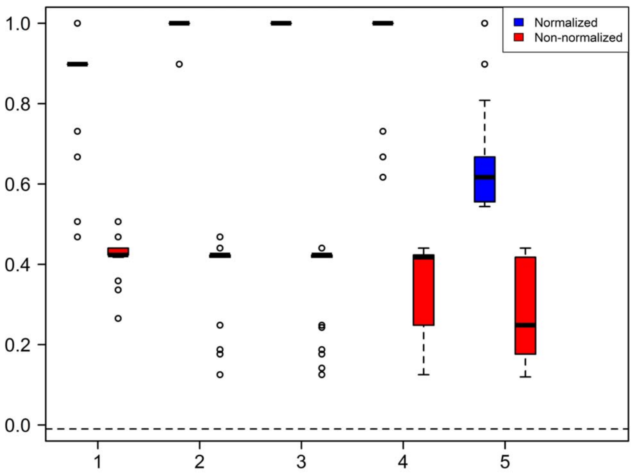

Figure 3. Boxplots of adjusted rand index for measuring the extent of agreement of clustering of the random networks using non-normalized f-Wiener indices versus normalized f-Wiener indices.
doi:10.1371/journal.pone.0078448.g003

networks with the number of nodes ranging from 500 to 950 with step of increment 50. Experiment 1 consists of 5 small, but similar, experiments. We enumerate these 5 small experiments as 1.1,…, 1.5. Subsection after experiments provides more details on how to generate these random networks. We then apply Steps 2 and 3 above to form two dendrograms: one using f-Wiener indices without normalization (Figure 2A) and the other dendrogram using normalized f-Wiener indices (Figure 2B). To quantify the classification of the two methods: with and without normalization, we adopt the commonly used Adjusted Rand Index (ARI) [38] for classification validation. ARI measures the accuracy of classification, and takes values between -1 and 1. The larger the ARI is, the better is the classification. The ARI for Figures 2A and 2B are respectively 0.18 and 0.56 for Experiment 1.5. Using normalized f-Wiener indices lead to a substantial improvement in the classification. We repeat Experiments 1.1 to 1.5 1000 times each. The boxplots of the ARI are shown in Figure 3. The means and standard deviations for these experiments are given in Table 1. They clearly demonstrate the superiority of classification using normalized f-Wiener indices.

Experiment 2: Hierarchical Clustering of Trees

The collection of trees to be classified consists of 10 paths (P_n), 10 stars (S_n), 10 brooms ($B_{n,\frac{n}{2}}$), 20 caterpillars ($C_{n,2}$ which is like a path, and $C_{n,\frac{n-10}{10}}$ which is like a star), and for n ranging from 500 to 950 with step of increment 50.

Figure 4 shows the two dendrograms. The ARI for Figures 4A and 4B are respectively 0.10 and 1.00. This demonstrates that using normalized f-Wiener indices provides much better accuracy for classification purposes. The result in this experiment is consistent with that of experiment 1.

Experiment 3: Hierarchical Clustering of Random Networks and Trees

The collection of networks consists of (i) networks generated by three random network models, namely, ER model, SF Model and 3-D geometric model; (ii) some trees such as paths, brooms, caterpillars, stars. Figure 5 shows the two dendrograms formed. And the ARI for Figures 5A and 5B are respectively 0.04 and 0.86.

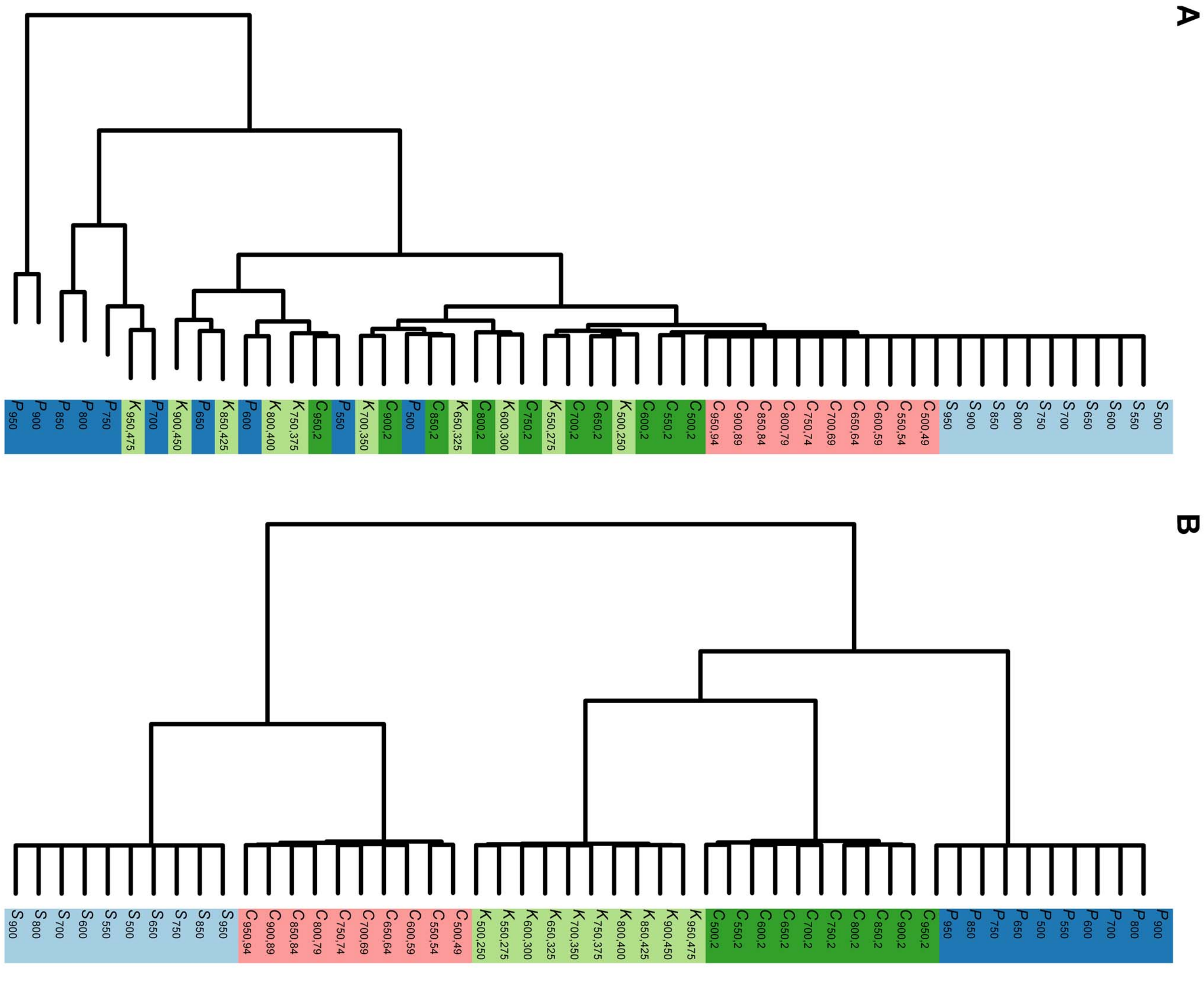

Figure 4. Hierarchical clustering of trees. Panel (A) shows the hierarchical clustering based on the f-Wiener indices (see Step 1 on page 6 for functions used). The adjusted rand index (ARI) is 0.1. Panel (B) shows the hierarchical clustering based on normalized f-Wiener indices. The ARI is 1. Trees used in the clustering consist of paths (P_n), stars (S_n), caterpillar-like trees ($C_{n,k}$), kites ($K_{n,k}$). Number of nodes $n = 500, 550, \ldots, 950$.
doi:10.1371/journal.pone.0078448.g004

Details on Generating Random Networks

We describe here in details on how to choose the networks generated by the three random network models in experiments 1 and 3.

Experiment 1 consists of 5 small, but similar, experiments which we label as Experiment 1.1, …, Experiment 1.5 which correspond to $p=0.01,\ldots,0.05$ respectively. Now we describe Experiment 1.5 in details.

ER Model

There are two parameters in the ER model, namely, n, the number of nodes, and p, the probability that an edge is formed between a pair of nodes. All edges are formed independently of each other. In Experiment 1.5, where $p=0.05$, we choose n ranging from 500 to 950 with step of increment 50. We generate an ER network using 'erdos.renyi.game' function available in the R package igraph [39]. If the network is connected, we keep it in $\mathcal{C}$ and denote it as ER_{500}. If not, then we repeat the function 'erdos.renyi.game' until a connected network is obtained. Similarly, $ER_{550},\ldots,ER_{950}$ are generated.

SF Model

We also construct ten SF networks by the function 'barabasi.-game' available in the R igraph package. We shall describe how to grow a SF network with 500 nodes for a given p, say $p=0.05$. The other 9 SF networks with $550,\ldots,950$ nodes are constructed in a similar manner. In 'barabasi.game' function, we set number of vertices 500, number of edges to be added in each time step $np/2$ rounded to the nearest integer, and the option to create a directed graph false.

Geometric Model

We generate ten 3-D geometric networks with $500,550,\ldots,950$ nodes. We shall describe how to construct one with 500 nodes as follows. The rest are constructed similarly. We first place 500 nodes in a unit cube uniformly and independently, then we

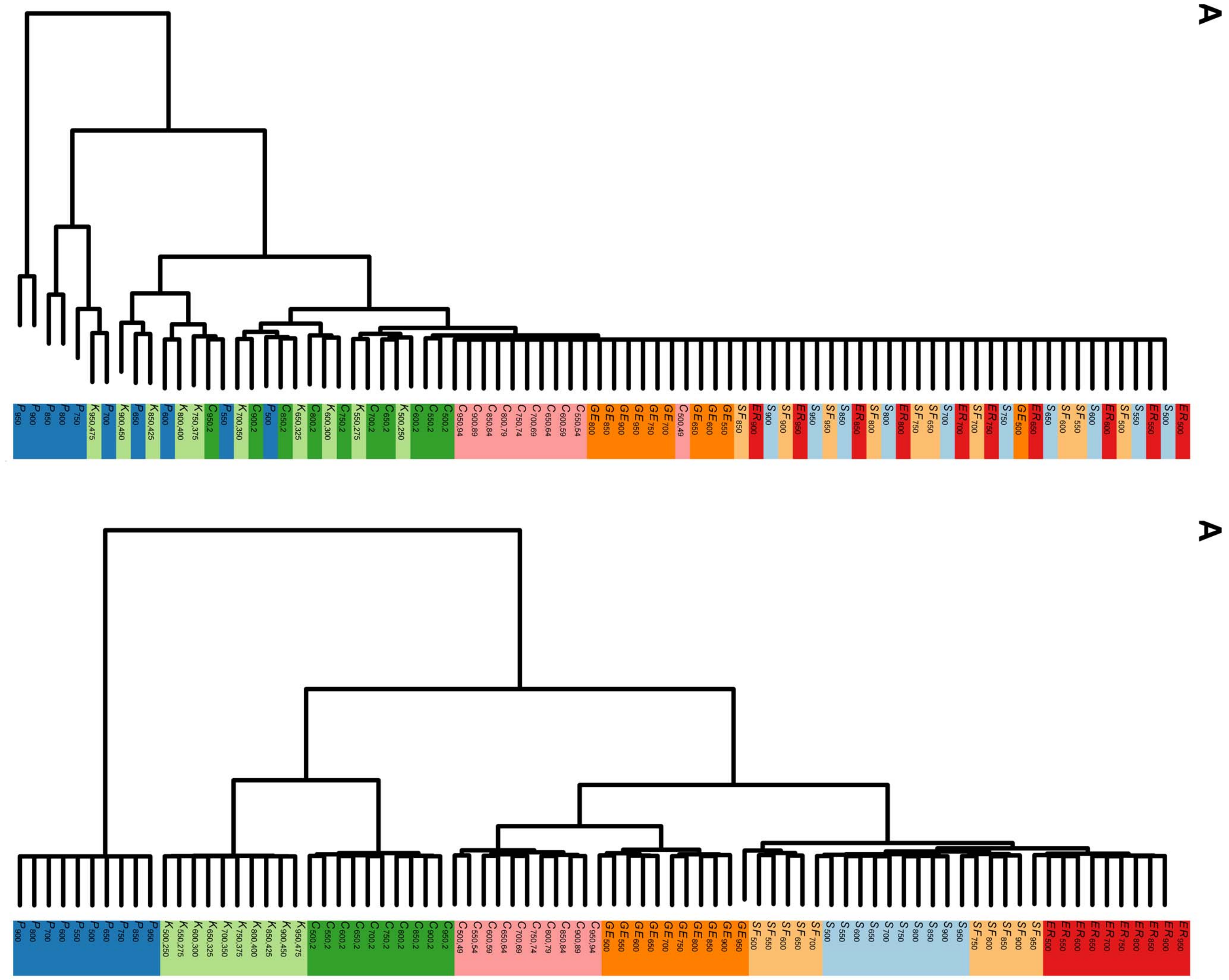

Figure 5. Hierarchical clusters of trees and graphs. Panel (A) shows the hierarchical clustering based on the f-Wiener indices (see Step 1 on page 6 for functions used). The adjusted rand index (ARI) is 0.04. Panel (B) shows the hierarchical clustering based on normalized f-Wiener indices, and ARI = 0.86. Trees used are paths (P_n), stars (S_n), caterpillar-like trees ($C_{n,k}$), kites ($K_{n,k}$). Graphs are generated by Erdos-Renyi (ER_n), scale-free (SF_n) and geometric (GE_n) random network models. The parameter, p, in the Erods-Renyi random graph equals to 0.05, number of nodes $n=500,550,\ldots,950$.
doi:10.1371/journal.pone.0078448.g005

compute all the $\binom{500}{2}$ pairwise distances and rank these distances in ascending order. We choose the top $100p\%$ of these pairwise distances and connect their corresponding nodes. If this network is connected, then we keep it in $\mathcal{C}$ and denote it by GE_{500}. Otherwise, we discard it, and repeat the above procedure until we get a connected network. The other networks $GE_{550},\ldots,GE_{950}$ are constructed similarly.

Conclusions

Wiener index and other Wiener type indices have been commonly applied in Chemometrics to associate structures and physicochemical properties of molecules. Recently, these indices are incorporated in quantifying complex networks as in QuACN [9] and NetCAD [40]. In this article, we first generalize Wiener index to a general functional form, called f-Wiener index. This f-Wiener index contains all well-known Wiener type indices as special cases such as Wiener index, Harary index, hyper Wiener index, compactness, and average efficiency. We provide a unifying method to identify the maximum and minimum over the set of simple connected graphs with n nodes, or the set of simple connected trees with n nodes (Theorems 1 and 2). Explicit sharp upper and lower bounds for Wiener index, Harary index, hyper Wiener index and the generalized index are deduced over networks (Corollary 5) and over trees (Corollary 6). Moreover, the maximizer and minimizer are characterized in Theorems 1 and 2. We believe these results are general and of independent interests.

Armed with these maximum and minimum values, we propose a normalized version of f-Wiener index over networks, and a similar version over trees. These normalized versions provide better interpretation of indices over networks of varying number of nodes than the non-normalized one. We conduct a number of experiments to compare the clustering performance using normalized f-Wiener indices with that of the non-normalized f-Wiener indices. The results of these experiments consistently demonstrate that using normalized versions improved clustering substantially. The normalized versions capture similar topological structures among networks with different number of nodes better. Our method of optimizing $W_f(G)$ can be easily extended to index of the form $\Phi(W_f(G))$ where Φ and f are monotone functions. For example, taking $\Phi(x)=1/x$ and $f(k)=\frac{2}{n(n-1)k}$ leads to $\Phi(W_f(G))=\frac{n(n-1)}{2\sum_{i<j}1/d(i,j)}$ which measures small-world behvaior of network G [8]. For other descriptors, it is of interest to study whether normalization is needed; if so, how best to normalize them; and to what extent normalization improve network comparison.

Observe that $W_f(G)=\sum_{r=1}^{\infty}f(r)n_r(G)=\sum_{r=0}^{\infty}[f(r+1)-f(r)]N_r(G)$ where we assume $f(0)=0$, $n_r(G)$ denotes the number of pairs of nodes in G with distance equals r, and $N_r(G)$ the number of pairs of nodes in G with distance greater than r. Since in most biological networks the number of nodes is large, one may normalize a scaled-version of $W_f(G)$ in terms of the asymptotic distribution of the N_r's under the assumption that the observed network G is generated by a given random network model $\mathcal{M}$. This will enable us to determine the likelihood that the observed network is generated by $\mathcal{M}$. Currently a fair amount of information about shortest paths in some network models is available in [41,42]. How to make use of these results seems like a worthwhile future project.

Supporting Information

Figure S1 Illustrating the choices of u_1,u_2 and u_3 in Lemma 2. Here T_1 has 5 nodes, T_2 3 nodes. We choose $u_1=3,u_2=5$ and $u_3=6$. Tree T is constructed by joining u_1 and u_3 while T' by joining u_2 and u_3. $D(T)$ and $D(T')$ are 8×8 matrices where the first 5 columns correspondent to the 5 nodes in T_1, and the last 3 rows correspondent to the 3 nodes in T_2.
(TIF)

Figure S2 Illustration of Lemma 3. Here $n=10,i=j=5,\ell=3$, $k=7$. From the counts of the distances above, it is clear that $(d'(u_3,v))_{v\in V(T')}\prec(d(u_1,v))_{v\in V(T)}$ and $D(T')\prec D(T)$.
(TIF)

Figure S3 Illustration of the subtree pruning and regrafting algorithm. Here T_0 is obtained from T first by deleting the edge (u_2,u_3) and then connecting u_1 and u_3. T_0 is proved to satisfy these properties: (i) $D(T)\prec D(T^0)$; (ii) $\Delta(T)-1\leq\Delta(T^0)\leq\Delta(T)$; and (iii) number of pendant nodes is one less than that of T.
(TIF)

Text S1 Detailed proof for Theorems 1–4.
(RAR)

Acknowledgments

The authors thank the anonymous reviewers for a careful reading of this article, helpful suggestions and bringing to their attention of some additional references.

Author Contributions

Conceived and designed the experiments: DT KPC. Performed the experiments: DT KPC. Analyzed the data: DT KPC. Wrote the paper: DC KPC. Performed the mathematical analysis and statistical analysis: DT KPC.

References

1. Vidal M, Cusick ME, Barabasi AL (2011) Interactome networks and human disease. Cell 144: 986–998.
2. Barabasi AL, Gulbahce N, Loscalzo J (2011) Network medicine: a network-based approach to human disease. Nature Reviews Genetics 12: 56–68.
3. Hu L, Huang T, Shi X, Lu WC, Cai YD, et al. (2011) Predicting functions of proteins in mouse based on weighted protein-protein interaction network and protein hybrid properties. PLoS One 6: e14556.
4. Delprato A (2012) Topological and functional properties of the small GTPases protein interaction network. PLoS One 7: e44882.
5. Resendis-Antonio O, Hernández M, Mora Y, Encarnación S (2012) Functional modules, structural topology, and optimal activity in metabolic networks. PLoS Computational Biology 8: e1002720.
6. Milenković T, Memišević V, Bonato A, Pržulj N (2011) Dominating biological networks. PloS One 6: e23016.
7. Junker BH, Schreiber F (2008) Analysis of biological networks. Wiley-Interscience, volume 2. 31–59.
8. Newman ME (2002) The structure and function of networks. Computer Physics Communications 147: 40–45.
9. Mueller L, Kugler K, Dander A, Graber A, Dehmer M (2011) QuACN: an R package for analyzing complex biological networks quantitatively. Bioinformatics 27: 140–141.
10. Wiener H (1947) Structural determination of paraffin boiling points. Journal of the American Chemical Society 69: 17–20.
11. Wiener H (1947) Correlation of heats of isomerization, and differences in heats of vaporization of isomers, among the paraffin hydrocarbons. Journal of the American Chemical Society 69: 2636–2638.
12. Plavšić D, Nikolić S, Trinajstić N, Mihalić Z (1993) On the harary index for the characterization of chemical graphs. Journal of Mathematical Chemistry 12: 235–250.

13. Randić M (1993) Novel molecular descriptor for structureproperty studies. Chemical Physics Letters 211: 478–483.
14. Zhang Y, Gutman I, Liu J, Mu Z (2012) q-analog of wiener index. Match: Communications in Mathematical and Computer Chemistry 67: 347.
15. Hosoya H (1988) On some counting polynomials in chemistry. Discrete Applied Mathematics 19: 239–257.
16. Brückler F, Došlić T, Graovac A, Gutman I (2011) On a class of distance-based molecular structure descriptors. Chemical Physics Letters 503: 336–338.
17. Balaban A (1982) Highly discriminating distance-based topological index. Chemical Physics Letters 89: 399–404.
18. Dehmer M, Mowshowitz A (2011) A history of graph entropy measures. Information Sciences 181: 57–78.
19. Dehmer M, Varmuza K, Borgert S, Emmert-Streib F (2009) On entropy-based molecular descriptors: Statistical analysis of real and synthetic chemical structures. Journal of Chemical Information and Modeling 49: 1655–1663.
20. Dehmer M (2008) Information processing in complex networks: Graph entropy and information functionals. Applied Mathematics and Computation 201: 82–94.
21. Todeschini R, Consonni V (2009) Molecular descriptors for chemoinformatics. Wiley-VCH.
22. Schmuck NS, Wagner SG, Wang H (2012) Greedy trees, caterpillars, and wiener-type graph invariants. Match-Communications in Mathematical and Computer Chemistry 68: 273.
23. Soltés L (1991) Transmission in graphs: a bound and vertex removing. Math Slovaca 41: 11–16.
24. Dobrynin A, Entringer R, Gutman I (2001) Wiener index of trees: theory and applications. Acta Applicandae Mathematicae 66: 211–249.
25. Gutman I, Linert W, Lukovits I, Dobrynin A (1997) Trees with extremal hyper-wiener index: Mathematical basis and chemical applications. Journal of Chemical Information and Computer Sciences 37: 349–354.
26. Resendis-Antonio O, Hernández M, Mora Y, Encarnación S (1931) Gráfok és mátrixok. Matematikai és Fizikai Lapok 38: 116–119.
27. Wagnera S, Wangb H, Zhangc XD (2013) Distance-based graph invariants of trees and the harary index. Filomat 27: 41–50.
28. Gutman I (1997) A property of the wiener number and its modifications. Indian journal of chemistry Sect A: Inorganic, Physical, Theoretical & Analytical 36: 128–132.
29. Todeschini R, Consonni V (2008) Handbook of molecular descriptors. Wiley-Vch.
30. Watts DJ, Strogatz SH (1998) Collective dynamics of 'small-world' networks. Nature 393: 440–442.
31. Gutman I, Popović L, et al. (1998) Graph representation of organic molecules Cayley's plerograms vs. his kenograms. Journal of the Chemical Society, Faraday Transactions 94: 857–860.
32. Ward Jr JH (1963) Hierarchical grouping to optimize an objective function. Journal of the American Statistical Association 58: 236–244.
33. R Development Core Team (2012) R: A Language and Environment for Statistical Computing. R Foundation for Statistical Computing, Vienna, Austria. URL http://www.R-project.org/. ISBN 3-900051-07-0.
34. Erdős P, Rényi A (1959) On random graphs. Publicationes Mathematicae Debrecen 6: 290–297.
35. Erdős P, Rényi A (1960) On the evolution of random graphs. Publications of the Mathematical Institute of the Hungarian Academy of Sciences 5: 17–61.
36. Barabási AL, Albert R (1999) Emergence of scaling in random networks. Science 286: 509–512.
37. Pržulj N, Corneil DG, Jurisica I (2004) Modeling interactome: scale-free or geometric? Bioinformatics 20: 3508–3515.
38. Rand WM (1971) Objective criteria for the evaluation of clustering methods. Journal of the American Statistical Association 66: 846–850.
39. Csardi G, Nepusz T (2006) The igraph software package for complex network research. InterJournal, Complex Systems 1695: 38.
40. Ren G, Liu Z (2013) NetCAD: a network analysis tool for coronary artery disease-associated PPI network. Bioinformatics 29: 279–280.
41. Barbour AD, Reinert G (2011) The shortest distance in random multi-type intersection graphs. Random Structures & Algorithms 39: 179–209.
42. Fronczak A, Fronczak P, Ho lyst JA (2004) Average path length in random networks. Physical Review E 70: 056110.
